# Extracellular matrix regulates lineage plasticity in prostate cancer through YAP/TEAD

**DOI:** 10.64898/2025.12.30.697072

**Published:** 2025-12-31

**Authors:** Teng Han, Zhen Sun, Matthew Lange, Y Zoe Cho, Patrick Mcgillivray, Maren Büttner, Nathaniel R Kastan, Subhiksha Nandakumar, Huiyong Zhao, Sanyukta Oak, Linda Fong, Wenfei Kang, Ning Fan, Jimmy Zhao, Nazifa Salsabeel, Harmanpreet Kaur, Ninghui Mao, Qing Chang, Eric Rosiek, Eric Chan, Murray Tipping, Nikolaus Schultz, Pierre-Jacques Hamard, Elisa DeStanchina, Dana Pe’er, Richard Koche, Zhenghao Chen, A James Hudspeth, Charles L Sawyers

**Affiliations:** 1. Human Oncology and Pathogenesis Program, Memorial Sloan Kettering Cancer Center, New York, NY 10065, USA; 2. Department of Medicine, Memorial Sloan Kettering Cancer Center, New York, NY 10065, USA; 3. Calico Life Sciences LLC, South San Francisco, CA 94080, USA; 4. Howard Hughes Medical Institute and Laboratory of Sensory Neuroscience, The Rockefeller University, New York, NY 10021, USA; 5. Marie-Josée and Henry R. Kravis Center for Molecular Oncology, Memorial Sloan Kettering Cancer Center, New York, NY 10065, USA; 6. Computational Oncology Service, Department of Epidemiology and Biostatistics, Memorial Sloan Kettering Cancer Center, New York, NY 10065, USA; 7. The Halvorsen Center for Computational Oncology, Memorial Sloan Kettering Cancer Center, New York, NY 10065, USA; 8. Antitumor Assessment Core Facility, Memorial Sloan Kettering Cancer Center, New York, NY 10065, USA; 9. Molecular Cytology Core Facility, Memorial Sloan Kettering Cancer Center, New York, NY 10065, USA; 10. Developmental and Regenerative Biology, Icahn School of Medicine at Mount Sinai, New York, NY 10029, USA; 11. Center for Epigenetics Research, Memorial Sloan Kettering Cancer Center, New York, NY 10065, USA; 12. Program for Computational and Systems Biology, Sloan Kettering Institute, Memorial Sloan Kettering Cancer Center, New York, NY 10065, USA; 13. Howard Hughes Medical Institute, Chevy Chase, MD 20815, USA

## Abstract

Treatment-related neuroendocrine prostate cancer (NEPC) is an increasingly frequent mechanism of resistance to androgen receptor pathway inhibitor (ARPI) therapy in prostate adenocarcinoma (PRAD). This lineage transition is dependent on upregulation of the NE-specifying transcription factor ASCL1, typically in a genetic background of *RB1* and *TP53* loss. Here we identify extracellular matrix-integrin-YAP1/TEAD signaling as a critical brake on NEPC lineage transition. Deletion of *Itgb1*, the shared B1 subunit required for collagen and laminin-mediated integrin activation, is sufficient to induce ASCL1 and NE lineage gene expression, by activating LATS1/2 kinases with subsequent inactivation of YAP1/TEAD signaling. Conversely, restoration of YAP1/TEAD signaling by pharmacological LATS1/2 inhibition, or by expression of constitutively active YAP1/TAZ mutants, prevents or reverts NEPC lineage transition. NOTCH and AR cooperate with YAP/TEAD to repress ASCL1, such that combined inhibition leads to complete reprograming of PRAD into NEPC *in vitro*, providing a dynamic platform to dissect the molecular events responsible for lineage transition over time. We find that lineage transition is accompanied by a redistribution of FOXA1 and TEAD cistromes from PRAD to NEPC-specific enhancers and requires the pioneering activity of FOXA1. Thus, extracellular matrix/integrin signaling in the PRAD tumor microenvironment restrains NE lineage plasticity, highlighting a potential path for pharmacological inhibitors in modulating treatment-induced lineage change.

## Introduction

Lineage plasticity - the ability of cancer cells to adopt alternative identities in response to environmental or therapeutic pressures - is increasingly recognized as a major mechanism of resistance to targeted therapies across multiple cancer types, including prostate cancer (1, 2). The mainstay of prostate cancer treatment is androgen deprivation therapy (ADT), to which most patients initially respond. However, resistance inevitably develops, leading to the emergence of castration-resistant prostate cancer (CRPC) (3). To counteract reactivated androgen receptor (AR) signaling in CRPC, next-generation AR pathway inhibitors (ARPIs) such as enzalutamide and abiraterone have become standard of care in CRPC and are now used in earlier treatment settings (4, 5). Despite improved overall survival, an unintended consequence of ARPI therapy is selective pressure that results in an increase in treatment-related neuroendocrine prostate cancer (NEPC) from prostate adenocarcinoma (PRAD), a lineage transition resembling EGFR-mutant lung adenocarcinomas that transition to small cell lung cancer (SCLC) following treatment with EGFR inhibitors (6, 7). Once this lineage switch occurs, NEPC is associated with poor clinical outcomes and is largely unresponsive to existing treatments (8), underscoring the need to understand the molecular events that initiate lineage plasticity, with the goal of preventing or delaying the transition.

Genomic loss of the *RB1* and *TP53* tumor suppressors is highly enriched in human NEPC (and in EGFR-mutant lung cancers that transition to SCLC) and is required for NEPC lineage transition in mouse models (9–12). However, the presence of these mutations alone is not sufficient to initiate lineage programing. For example, *Rb1*/*Trp53* deficient mouse prostate organoids retain a luminal/basal PRAD lineage when propagated *in vitro* but undergo lineage transition after *in vivo* transplantation, suggesting a critical role for the tumor microenvironment (TME) (9, 13). Additionally, genomically annotated cohorts of CRPC and EGFR-mutant lung cancer reveal that not all adenocarcinomas with *RB1* and *TP53* loss undergo lineage transition in response to therapy (12, 14). The fact that treatment-induced lineage transitions can be reversible further suggests that epigenetic programs likely play a critical role (15–17). Current evidence supports a model in which *RB1* and *TP53* loss establishes a chromatin state permissive for lineage switching, but additional tumor cell-extrinsic signals from the TME are needed to initiate and sustain lineage plasticity. Here we explore the nature of these TME cues and how they integrate with tumor-intrinsic programs.

## Results

As a first step, we confirmed prior work (9, 13) demonstrating that genetically engineered murine organoids with *Rb1* and *Trp53* loss coupled with *cMyc* overexpression (RPM) undergo a PRAD to NEPC transition following orthotopic transplantation into mice but fail to do so in organoid culture, as indicated by the absence of staining for the NE lineage marker *Ascl1* (Fig. S1A–B). We extended this phenotype to two additional *Rb1*/*Trp53*-deficient genotypes: *Rb1*^*−/−*^*; Trp53*^*−/−*^*; Pten*^*−/−*^ (TKO) and *Rb1*^*−/−*^*; Trp53*^*−/−*^*; Pten*^*−/−*^*; cMyc*^+^ (TKOM) (Fig. S1A–B). These findings document that conventional organoid culture systems fail to fully replicate the *in vivo* TME necessary for adenocarcinoma-to-NE transition.

### Relative depletion of stroma and extracellular matrix in NEPC

**Fig. 1.**
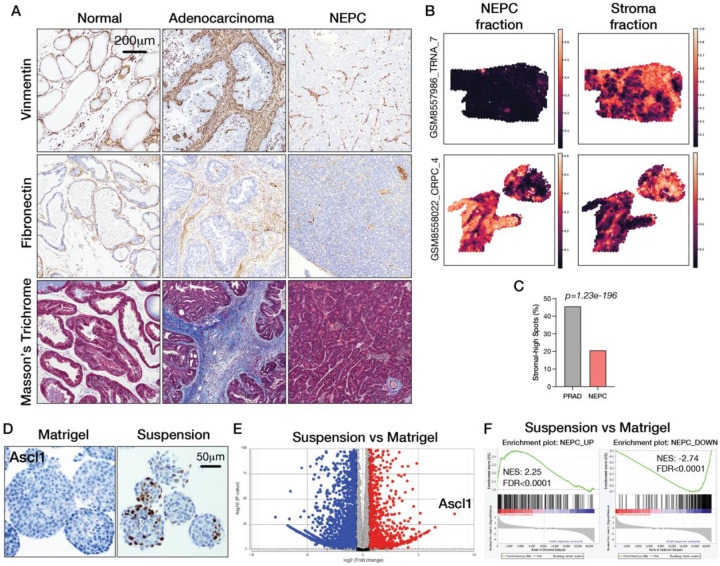
Removal of ECM activates Ascl1 expression. (A) IHC of Vimentin and Fibronectin, along with Masson’s trichrome staining of normal prostate tissue, adenocarcinoma regions, and neuroendocrine regions from TKO tumors. (B) Deconvolved NEPC theta estimates and corresponding stromal theta estimates across two human CRPC specimens. Spatial maps highlight the distribution of NEPC and fibroblast-like content across Visium spots. (C) Proportion of spots classified as both tumor-high and stroma-high for PRAD compared to NEPC. (D) ASCL1 IHC of TKOM organoids cultured in Matrigel versus suspension conditions. (E) Volcano plot showing differentially expressed genes from RNA-seq analysis comparing TKOM organoids cultured in suspension versus Matrigel. (F) Gene set enrichment analysis (GSEA) of RNA-seq data from TKOM organoids in suspension versus Matrigel culture using previously reported NEPC signatures derived from GEMMs (17).

To search for TME factors responsible for this lineage transition, we analyzed histologic sections from wild-type normal prostate tissue, as well as PRAD and NEPC regions from the PtRP (*Pten*^*−/−*^, *Rb1*^*−/−*^, *Trp53*^*−/−*^, *Pb-Cre*) genetically engineered mouse model (GEMM) (17). Strikingly, we observed that v*imentin*-positive fibroblasts, which are sparse in normal prostate tissue but expanded in PRAD regions, are relatively depleted in NEPC regions. Similarly, extracellular matrix (ECM) proteins, such as fibronectin and collagen—primarily produced by fibroblasts—are scarce in normal prostate tissue, abundant in PRAD and depleted in NEPC (Fig. 1A). Tumor-associated fibroblasts and collagen were also abundant in the TKO and RPM orthotopic transplantation models at the PRAD stage (2 weeks post-transplantation) but depleted following the transition to NEPC (16 weeks for TKO and 7 weeks for RPM) (Fig. S1C).

To explore whether a similar depletion of fibroblasts is observed in human NEPC, we analyzed a recently published prostate cancer cohort profiled using 10X Visium spatial transcriptomics (18) for stromal content in regions of PRAD versus NEPC. Using BayesPrism deconvolution (19, 20) to estimate the fractional contribution of PRAD, NEPC and stromal cells within each Visium spot (based on estimated cell type fraction >0.25, see methods), we identified ten specimens with sufficient NEPC content for inclusion in pooled analyses. Among PRAD-high spots, 45.7% had high stromal content (34,716/75,937) compared to 20.7% of NEPC-high spots (771/3,719) (Fig 1B–C, S1D). Taken together with the mouse histology, the results suggest that NEPC may preferentially emerge or expand in stroma-poor environments.

### ECM withdrawal initiates PRAD to NEPC lineage transition

Organoids are grown in Matrigel, an ECM preparation containing laminin (60%) and collagen IV (30%) that replicates the stroma-rich TME of PRAD. Because the NEPC transition is associated with depletion of stroma, we considered the possibility that Matrigel prevents the NEPC transition from occurring *in vitro*. To test this hypothesis, we removed Matrigel by growing TKOM organoids in suspension and monitored *Ascl1* expression as an early marker of NE lineage differentiation. *Ascl1* is not expressed in the luminal adenocarcinoma lineage but is essential for commitment to a neuroendocrine (NE) fate (9, 13). Notably, *Ascl1* protein expression was detected by IHC in a subset of cells within individual organoids after 30 days of suspension culture (Fig. 1D). To explore the kinetics of *Ascl1* induction, we performed a time course experiment and documented induction of *Ascl1* mRNA by RT-PCR after 7 days in suspension, with increased levels after 14 days and reaching peak levels at 30 days (Fig. S2A). Of note, *Ascl1* scored as the top upregulated gene in suspension culture by bulk RNA sequencing (RNA-seq) among other NEPC-associated TFs, notably *Foxa2*, *Insm1*, and *Sox2* (Fig. 1E, S2B). Furthermore, gene signatures associated with NEPC were enriched in suspension culture while those downregulated in NEPC were depleted (Fig. 1F). Thus, removal of Matrigel from TKOM organoids is sufficient to activate a transcriptional program resembling NEPC.

To explore the dynamics of this phenotypic shift at a single cell level, we performed multiome (scRNA and scATAC) sequencing of TKOM organoids grown in Matrigel, or in suspension culture for 2 days or 20 days. *Ascl1* mRNA was detectable after 20 days but not in all cells (Fig. S2C, S2D), consistent with the IHC data, and closely matched with changes in chromatin accessibility at the Ascl1 locus (Fig. S2E). *Foxa2* and *Insm1* were also detectable at day 20 in a subfraction of the Ascl1+ cells (Fig. S2F, S2G), suggesting that Ascl1 expression may precede expression of other NE genes, consistent with its role as a NE lineage master regulator.

### ECM-integrin signaling maintains PRAD lineage

**Fig. 2.**
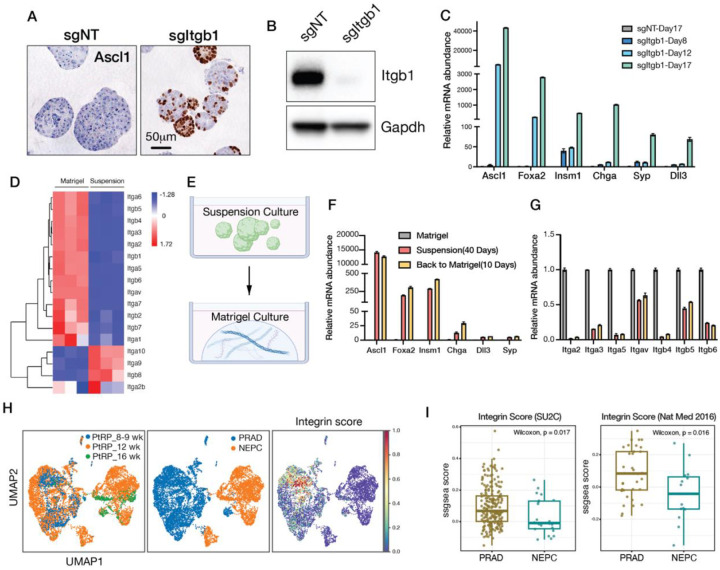
Loss of ECM-integrin signaling promotes PRAD to NEPC lineage transition. (A) ASCL1 IHC of TKOM organoids 17 days after electroporation with control sgRNA (sgNT) or sgRNA targeting Itgb1 (sgItgb1). (B) Western blot confirming ITGB1 knockout in TKOM organoids. (C) Quantitative reverse-transcription PCR (qRT-PCR) analysis showing expression of NE markers at day 8, 12 and 17 following *Itgb1* deletion. Error bars represent ±SEM, n=3. (D) Heatmap showing differential expression of integrin genes in TKOM organoids cultured in Matrigel versus suspension. (E) Schematic of the replating experiment. (F) qRT-PCR of neuroendocrine markers in TKOM organoids cultured in Matrigel, suspension conditions and replating back to Matrigel. Error bars represent ±SEM, n=3. (G) qRT-PCR showing the expression of multiple integrin genes in TKOM organoids cultured in Matrigel, suspension conditions and after replating back in Matrigel. Error bars represent ±SEM, n=3. (H) scRNA-seq analysis of PtRP (TKO) GEMMs showing reduced integrin gene expression score in NEPC compared to PRAD. (I) Box plot demonstrating reduced integrin gene expression score in NEPC (CRPC-NE) compared to PRAD (CRPC-adeno) in two published CRPC patient cohorts. The integrin gene expression score represents the mean expression of all integrin genes.

While the suspension culture experiments implicate components of Matrigel as inhibitors of NE lineage transition in organoid culture, interpretation is confounded by the fact that prolonged growth of epithelial cells in suspension culture can enrich for other phenotypes such as acquisition of stem-like properties (21). The primary constituents of Matrigel, laminin and collagen IV, signal through distinct alpha integrin receptors but share a common beta receptor subunit, integrin beta-1 (ITGB1) (22). To ask if the induction of *Ascl1* seen in suspension culture is a consequence of impaired integrin signaling, we deleted *Itgb1* in TKOM organoids. Remarkably, *Itgb1* knockout replicated the mosaic pattern of ASCL1 protein expression observed in suspension culture but now in organoids grown in Matrigel (Fig. 2A–B). *Itgb1* loss also resulted in upregulation of *Foxa2* and *Insm1*, as well as the NE markers C*hga, Syp* and *Dll3* (Fig. 2C). These findings establish that ECM-integrin signaling in prostate organoids is critical to maintain the PRAD lineage state and acts as a brake on the NEPC transition *in vitro*.

We next considered whether and how ECM-integrin signaling might be disrupted in the *in vivo* setting. To model disrupted ECM signaling, we examined gene expression changes seen after transfer of TKOM organoids to suspension culture and noticed that 7 alpha integrins (*Itga1,2,3,5,6,7,v*) and 5 beta integrins (*Itgb1,4,5,6,7*) were significantly downregulated (Fig. 2D). To determine if this level of downregulation has functional consequences, we replated suspension cells back into Matrigel to reactivate integrin signaling but found that NE marker expression remained stable (Fig. 2E–F). Thus, activation of the NE lineage program that follows loss of ECM-integrin signaling cannot be reversed by reengagement with ECM, likely due to sustained downregulation of integrin expression (Fig. 2G). Using an integrin score that quantifies expression of multiple integrin genes, we also observed significantly reduced expression in NEPC versus PRAD tumor cells in PtRP mice (Fig. 2H) and in human CRPC cohorts with PRAD and NEPC patients (Fig. 2I) (12, 23).

To summarize, ECM-integrin signaling restricts the PRAD to NEPC lineage transition in organoid culture. Once this signal is disrupted, loss of integrin expression by tumor cells precludes reversion back to a PRAD lineage state, even if abundant ECM is present. These findings support a model where the abundant stromal population associated with the PRAD state *in vivo* restrains lineage plasticity. Consistent with this model, NEPC emerges in regions with a relative paucity of stromal cells.

### YAP1/TAZ/TEAD suppresses Ascl1 induction and NE lineage transition

**Fig. 3.**
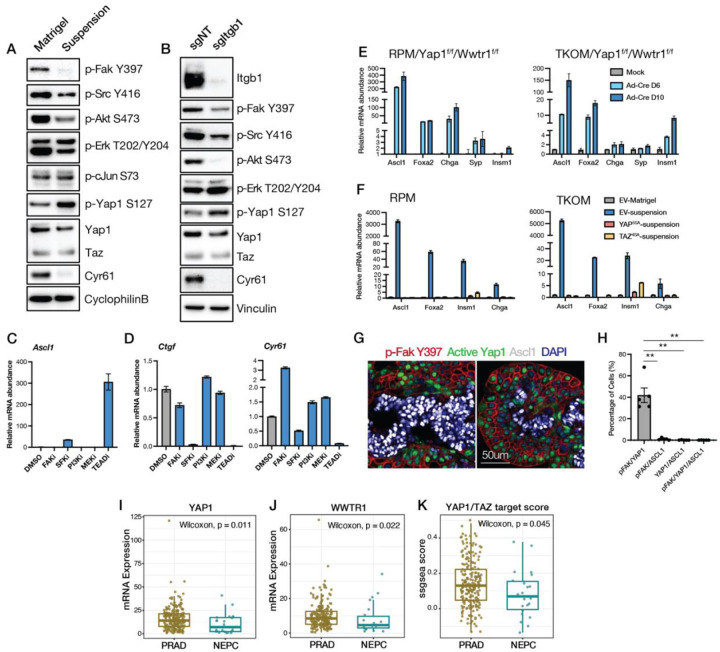
YAP/TEAD activation blocks Ascl1 induction. (A-B) Western blot analysis of representative kinases, substrates and target genes downstream of integrin downstream in TKOM organoids in Matrigel or after one day in suspension culture (A) or two days after *Itgb1* knockout (B). (C) qRT-PCR for *Ascl1* in TKOM organoids after treatment with pharmacologic inhibitors of the indicated targets. FAKi: 5uM PF573228; SFKi: 5uM Dasatinib; PI3Ki: 2uM GDC0941; MEKi: 1uM AZD6244; TEADi: 1uM IAG933. Error bars, ±SEM, n=3. (D) qRT-PCR of the YAP1/TAZ target genes *Ctgf* and *Cyr61* in TKOM organoids after treating with the indicated inhibitors. Error bars, ±SEM, n=3. (E) qRT-PCR of NE genes in RPM/*Yap1*^*f/f*^*/Wwtr*^*f/f*^ and TKOM/*Yap1*^*f/f*^*/Wwtr*^*f/f*^ organoids 6 days and 10 days following Adeno-Cre (Ad-Cre) infection. Error bars, ±SEM, n=3. (F) qRT-PCR of NE markers in RPM and TKOM organoids overexpressing EV, constitutive YAP^5SA^ or TAZ^4SA^, cultured in Matrigel or in suspension for 14 days. Error bars, ±SEM, n=3. (G-H) Multiplex IF of phospho-FAKY397, active-YAP1 and ASCL1 of tumor sections from 12-week-old TKO GEMM (G) with quantification (H). Error bars, ±SEM, n=3, **p<0.005. (I-K) Box plot demonstrating reduced expression of *YAP1* and *WWTR1*, as well as decreased YAP/TAZ target score, in NEPC (CRPC-NE) compared to PRAD (CRPC-adeno) tumors from the SU2C cohort.

To elucidate which pathways downstream of integrin engagement suppress the induction of Ascl1 expression, we first examined canonical kinase signaling events associated with integrin activity (24). pFAK, pSRC and pAKT were attenuated when cells were cultured in suspension or following *Itgb1* knockout, confirming that the circuitry of integrin signaling that is well defined in other cell types is conserved in prostate organoid culture (Fig. 3A–B). We also examined the status of the YAP1/TAZ/TEAD (HIPPO) pathway (hereafter called YAP/TEAD), a target of integrin signaling linked to mechanical sensing (ECM stiffness) (25). Loss of integrin engagement resulted in reduced YAP/TEAD signaling, as measured by increased pYAP1 (a substrate for LATS1/2 kinase) and reduced expression of the YAP/TEAD target gene CYR61 (Fig. 3A–B). To determine which, if any, of these signaling events are involved in *Ascl1* upregulation, we used a panel of small molecule inhibitors targeting FAK, SRC, PI3K, MEK or the YAP/TEAD complex to test if any might reproduce the *Ascl1* induction phenotype seen with *Itgb1* KO. Of the kinase inhibitors, only the SRC family inhibitor dasatinib gave a reproducible (~30-fold) increase in *Ascl1* expression. More striking, however, was a ~300-fold increase in *Ascl1* seen with IAG933, which selectively disrupts the YAP/TEAD protein-protein interaction (Fig. 3C) (26). IAG933 (hereafter called TEADi) also blocked expression of the YAP/TEAD target gene *Ctgf* and *Cyr61* (~90% decrease), as did dasatinib (~50% decrease), consistent with the known role of SRC kinases in activating YAP/TEAD through LATS1/2 inhibition (Fig. 3D) (27).

To build on the pharmacologic evidence implicating YAP/TEAD as a critical regulator of *Ascl1* induction, we turned to genetic experiments using gain and loss of function approaches. First, we generated RPM and TKOM mouse prostate organoids in a *Yap1*^*fl/fl*^/*Wwtr1*^*fl/fl*^ background (the *Wwtr1* gene encodes TAZ) to ask whether deletion of these TEAD co-activators would activate NE gene expression. We observed >100-fold induction of *Ascl1* and upregulation of other NE genes 6–10 days after co-deletion of *Yap1*/*Wwtr1* by adenoviral Cre infection (Fig. 3E), as well as the expected loss of YAP/TEAD target gene expression (*Cyr61*, *Ctgf*, *Axl, Ptpn14*) (Fig. S3A). *Yap1*/*Wwtr1* co-deletion also resulted in downregulation of several alpha (*Itga2*, *Itga5*, *Itgav*) and beta integrin genes (*Itgb4, Itgb5, Itgb6*) (Fig. S3B), mirroring the loss of integrin expression seen earlier when *Yap1*/*Wwtr1* intact organoids were placed in suspension culture (Fig. 2D). Thus, YAP/TEAD is a key regulator of integrin expression in this context. Conversely, expression of a constitutively active mutant of either YAP (YAP^5SA^) or TAZ (TAZ^4SA^), both of which are resistant to inactivation by LATS1/2 phosphorylation, blocked the induction of *Ascl1* mRNA and other NE markers (*Foxa2*, *Insm1*, *Chga*) when RPM and TKOM organoids were placed in suspension culture, while maintaining YAP/TAZ target gene expression (*Cyr61*, *Ctgf*, *Axl, Ptpn14*) (Fig. 3F, S3C).

Collectively, the organoid experiments provide evidence that ECM-integrin engagement reinforces PRAD lineage identity through YAP/TEAD activation. As discussed earlier, PRAD to NEPC lineage plasticity *in vivo* is associated with a transition from a stroma rich (high ECM) to a stroma poor (low ECM) TME (Fig. 1A–B). To determine whether these TME changes are also linked to YAP/TEAD activity *in vivo*, we performed multiplex immunofluorescence (IF) on prostate tissue sections from 9-week-old PtRP mice, when NEPC first starts to emerge from PRAD. Using phosphoFAK (pFAK) as a readout for ECM-integrin engagement, we found that loss of pFAK in tumor cells was highly correlated with reduced nuclear YAP1 staining and gain of ASCL expression as measured by multiplex immunofluorescence (*p*<0.005) (Fig. 3G–H). Furthermore, YAP/TEAD activity in human CRPC cohorts (via RNA signatures) was significantly reduced in NEPC compared to PRAD (Fig. 3I–3K), consistent with scRNA-seq data from a smaller CRPC cohort (28). In sum, evidence from organoids, GEMMs and human CRPC supports a model whereby ECM-integrin engagement maintains the PRAD lineage through sustained YAP/TAZ/TEAD signaling.

### LATS inhibition impairs acquisition and maintenance of neuroendocrine state

**Fig. 4.**
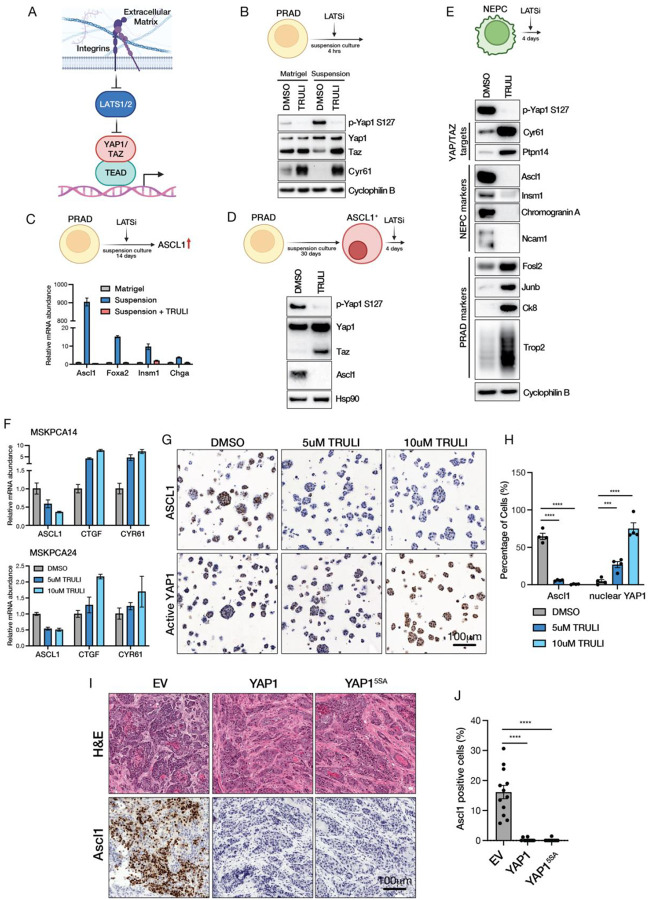
LATS inhibition restores YAP/TEAD activity and reverses NE lineage transition. (A) Cartoon showing ECM-Integrin inhibits LATS1/2 to activate YAP1/TAZ/TEAD. (B) Upper: Schematic of the experimental design. Lower: Western blot analysis of indicated proteins in TKOM organoids cultured in Matrigel or suspension, treated with 5uM TRULI for 4 hours. (C) Upper: Schematic of the experimental design. Lower: qRT-PCR of NE genes (*Ascl1*, *Foxa2*, *Insm1*, and *Chga*) in TKOM organoids cultured in Matrigel, suspension, or suspension plus 5uM TRULI for 14 days. Error bars represent ±SEM, n=3. (D) Upper: Schematic of the experimental design. Lower: Western blot analysis of indicated targets in RPM PRAD organoids cultured in suspension for 30 days followed by treatment with 5uM TRULI for 4 days. (E) Upper: Schematic of the experimental design. Lower: Western blot analysis of indicated targets in RPM NE organoids following treatment with 5uM TRULI for 4 days. (F) qRT-PCR analysis of ASCL1 and YAP1/TAZ targets (CTGF and CYR61) in MSKPCA14 and MSKPCA24 patient-derived organoids treated with DMSO, 5uM TRULI, 10uM TRULI for 10 days. Error bars represent ±SEM, n=3. (G) ASCL1 and YAP1 IHC in MSKPCA14 patient-derived organoids treated with DMSO, 5uM TRULI, or 10uM TRULI for 10 days. (H) Quantification of the percentage of ASCL1 positive cells and nuclear YAP1 positive cells. Error bars represent ±SEM, n=4, ***p<0.0005, ****p<0.0001. (I) Representative H&E images and ASCL1 IHC images of RPM transplantation tumors overexpressing EV or wild-type YAP1 or YAP15SA. (J) Quantification of the percentage of Ascl1 positive cells in (I). Error bars represent ±SEM, n=12, ****p<0.0001.

Earlier we showed that the PRAD to NEPC lineage transition following loss of ECM-integrin engagement cannot be reversed by re-exposure to ECM - likely due to loss of integrin expression (Fig. 2E–G). However, the fact that YAP1/TEAD activity is regulated by LATS1/2 kinase activity (YAP1 and TAZ phosphorylation by LATS1/2 results in cytoplasmic retention and inability to bind TEAD (29)) led us to ask whether the reduced YAP/TEAD activity caused by loss of ECM-integrin engagement could be restored through LATS kinase inhibition (Fig. 4A). Indeed, treatment of TKOM organoids with the LATS1/2 kinase inhibitor TRULI (30) (hereafter called LATSi) blocked YAP1 phosphorylation induced by suspension culture and restored expression of canonical YAP/TEAD target genes such as CYR61 (Fig. 4B). We then continued treatment for 14 days and found that LATSi prevented induction of *Ascl1* and other NE markers (*Foxa2*, *Insm1*, *Chga*) (Fig. 4C, S4A). Importantly, induction of ASCL1 was reversed when LATSi was added after prolonged growth in suspension culture (Fig. 4D).

We next asked whether LATSi can reverse the fully established NEPC tumor phenotype that develops *in vivo* by establishing ASCL1+ tumoroids from NEPC tumors that developed following orthotopic transplantation of RPM and TKO organoids (see methods for details) (Fig S4B–E). Again, LATSi blocked YAP1 phosphorylation and restored YAP/TEAD activation, as shown by induction of target genes such as CYR61 and PTPN14. Remarkably, after 4 days of treatment, these NEPC tumoroids had near complete loss of ASCL1, INSM1 and CHGA expression and gained expression of PRAD lineage markers such as CK8, TROP2 and the AP1 complex proteins FOSL2 and JUNB (Fig. 4E), providing proof of concept for reversal of an established NEPC transition back to a PRAD state by pharmacological restoration of YAP/TEAD activity.

Having shown that the PRAD to NEPC lineage transition can be blocked or reversed by LATSi in mouse tumoroid models, we asked whether this reversion extends to human NEPC. Using patient-derived organoids (PDOs) established from CRPC patients with NEPC (31, 32), we found that LATSi activated YAP/TEAD target gene expression (*CYR61*, *CTGF*) in a concentration-dependent manner, confirming the same pathway is intact in human CRPC models (Fig. 4F). Treatment with LATSi robustly restored YAP1 nuclear protein expression, coupled with a reduction in the percentage of ASCL1 expressing cells from >60% to <5% (Fig. 4G–H), further credentialling LATS1/2 kinase as a potential therapeutic target to prevent or reverse PRAD to NEPC lineage plasticity.

Unfortunately, we were unable to test this hypothesis in mice using TRULI or the chemically related LATSi TDI-011536 (33) due to rapid *in vivo* clearance and full recovery of pYAP in prostate tissue 4 hours after drug administration (Fig S4F). We therefore turned to a genetic approach, using the previously described constitutively active YAP1^5SA^ allele (Fig 3F) but now in a doxycycline (Dox) inducible format such that we could enforce YAP activation in RPM organoids during the time interval when the PRAD to NEPC transition occurs. Remarkably, sustained induction of YAP1^5SA^ (or wild-type YAP1) starting 2 weeks after transplantation prevented the appearance of any detectable ASCL1+ cells in PRAD tumors that emerged 21 days later, whereas tumors that developed from RPM organoids transduced with the Dox empty vector control (EV) had abundant regions of ASCL1+ NEPC (Fig 4I–J), indicating that sustained YAP/TEAD activation can prevent PRAD to NEPC lineage plasticity *in vivo*.

### Cooperativity of YAP1/TEAD, NOTCH and AR signaling in PRAD lineage maintenance

**Fig. 5.**
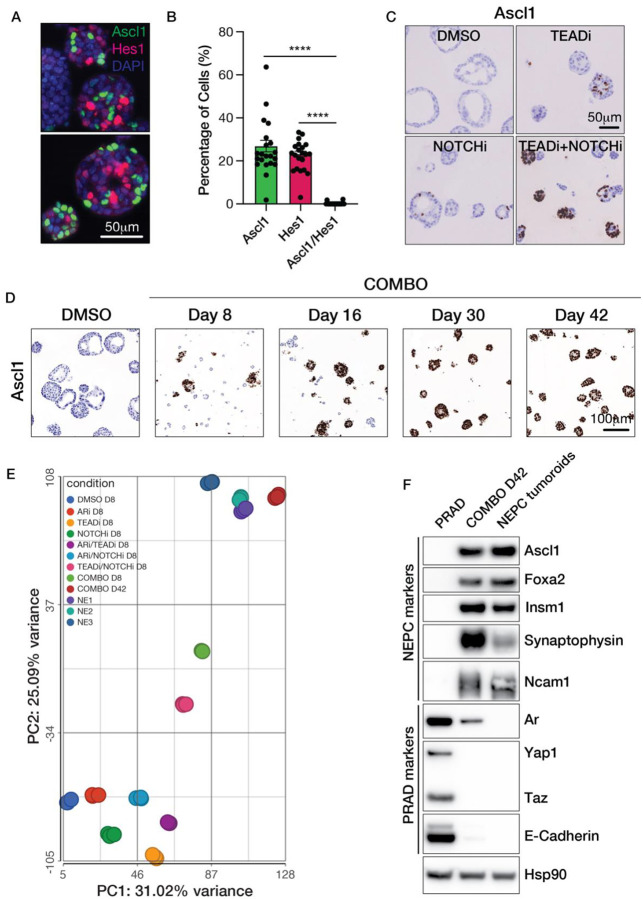
Reconstitution of PRAD to NEPC lineage transition *in vitro*. (A and B) Multiplex IF staining for ASCL1, HES1 and DAPI in TKOM organoids cultured in suspension (A) and quantified (B). Error bars, ±SEM, n=20, ****p<0.0001. (C) ASCL1 IHC in TKO PRAD organoids with DMSO, TEADi (1uM IAG933), NOTCHi (10uM DAPI) or a combination of TEADi and NOTCHi for eight days. The scale bar represents 50um. (D) ASCL1 IHC in TKO PRAD organoids treated with COMBO treatment over the indicated time course. The scale bar represents 100um. (E) Principal component analysis (PCA) of RNA-seq data from TKO PRAD organoids subjected to the indicated treatments. (F) Western blot of NEPC and PRAD lineage markers in TKO PRAD organoids, TKO PRAD organoids treated with COMBO for 42 days (COMBO D42), and NEPC tumoroids.

The above data provide evidence that disruption of YAP/TEAD activity (through suspension culture, *Itgb1* deletion or pharmacologic blockade of YAP/TEAD binding) promotes NE lineage transition. However, *in situ* analysis of ASCL1 protein expression reveals a mosaic pattern with all three methods of YAP/TEAD perturbation (Figs. 1D, 2A, 5C) suggesting that other regulatory pathways may play a role. In addition, the mRNA levels of *Ascl1* and other NE markers (*Foxa2*, *Insm1*, *Ncam*) achieved in organoids following YAP/TEAD perturbation do not reach the level seen in NEPC tumors that develop in organoids transplanted *in vivo* (Fig. S5A).

The mosaic pattern of ASCL1 expression is reminiscent of segmental patterns of protein expression seen in normal tissue development, often a consequence of cell-cell communication through signaling pathways such as NOTCH. Furthermore, NOTCH is known to suppress NE differentiation across various tissues, including in models of NEPC and ASCL1+ small cell lung cancer (34–36). To determine if NOTCH plays a similar role in prostate organoids, we first performed dual IF staining of ASCL1 and the NOTCH target HES1 in TKO organoids grown in suspension culture. ASCL1 and HES1 were robustly expressed in a mutually exclusive pattern in all organoids examined (Fig. 5A–B). To test the hypothesis that NOTCH activation prevents *Ascl1* induction in the TKO model, we treated organoids with the gamma secretase inhibitor DAPT (hereafter called NOTCHi) (37) alone or in combination with TEADi for 8 days. NOTCHi alone resulted in a mosaic pattern of ASCL1 expression, reminiscent of that seen with TEADi albeit less pronounced. However, combination treatment with NOTCHi and TEADi resulted in intense ASCL1 expression in nearly all the cells within each organoid (Fig. 5C), indicative of cooperativity between NOTCH and YAP/TEAD activation in suppressing ASCL1. Since ARPI therapy typically precedes the emergence of NEPC, we also measured the consequences of AR inhibition. Addition of enzalutamide plus DHT withdrawal (hereafter called ARi) to NOTCHi and TEADi treatment (hereafter called COMBO) further enhanced *Ascl1* induction (Fig. S5B) coupled with a progressive increase in the percentage of organoids that now uniformly express ASCL1 over 30–42 days (Fig. 5D).

### Complete reconstitution of PRAD to NEPC lineage transition *in vitro*

The robust and homogeneous pattern of ASCL1 expression seen after 6–7 weeks of COMBO treatment suggests PRAD organoids may undergo progressive reprograming to a complete NEPC lineage state. To address this possibility, we compared the transcriptomes of PRAD organoids treated with vehicle control (DMSO), single inhibitors (TEADi, NOTCHi or ARi), doublet combinations (TEADi /NOTCHi, TEADi/ARi, NOTCHi/ARi) and COMBO in a time course experiment. Using the time course of ASCL1 expression as a guide (Fig. 5D), we harvested organoids at 8 days to capture an intermediate state of lineage reprogramming and at 42 days to represent the final NE lineage state achieved *in vitro*. As an additional control, we included three independently derived NEPC tumoroids (NE1–3) to compare the NE state generated by *in vitro* reprogramming with the fully reprogramed NEPC state that develops *in vivo*.

Using principal component analysis (PCA) to display the entirety of the transcriptomic data, we found that each single treatment and both ARi doublet treatments clustered relatively closely with the DMSO control, indicative of modest cell state changes when assessed at the level of the whole transcriptome (Fig. 5E). In stark contrast, organoids treated for 42 days with COMBO clustered at the other extreme of the PCA plot, together with all three NE tumoroids, suggesting that sustained COMBO treatment of PRAD organoids *in vitro* replicates the full NEPC transition seen *in vivo*. The 8-day timepoint for the TEADi/NOTCHi doublet and COMBO treatments were similarly revealing because both cluster near the center of the PCA plot, suggestive of a transition state between PRAD (Fig. 5E, bottom left) and NEPC (Fig 5E, upper right). To verify that the extremes of the PCA plot do indeed reflect the PRAD and NEPC states respectively, we generated heatmaps using previously reported PRAD and NEPC mRNA signatures (from the PtRP GEMM) (17) across all treatment conditions and timepoints. Indeed, the NE signature is high in NE tumoroids and COMBO but absent in DMSO and all single inhibitor (and ARi combo) treatments. Conversely, the PRAD signature is high in DMSO and all single inhibitor (and ARi combo) treatments but low in NE tumoroids and COMBO (Fig. S5C–D). As further confirmation, expression of NE markers at the protein level (ASCL1, FOXA2, INSM1) after 42 days of COMBO are comparable to those seen in NE tumoroids, as well as loss of PRAD markers such as E-cadherin (Fig. 5F). In sum, these data establish an organoid platform for complete reprogramming from PRAD to NEPC through combined and sustained inhibition of TEAD, NOTCH and AR.

### FOXA1 is essential for the PRAD to NEPC lineage transition

**Fig. 6.**
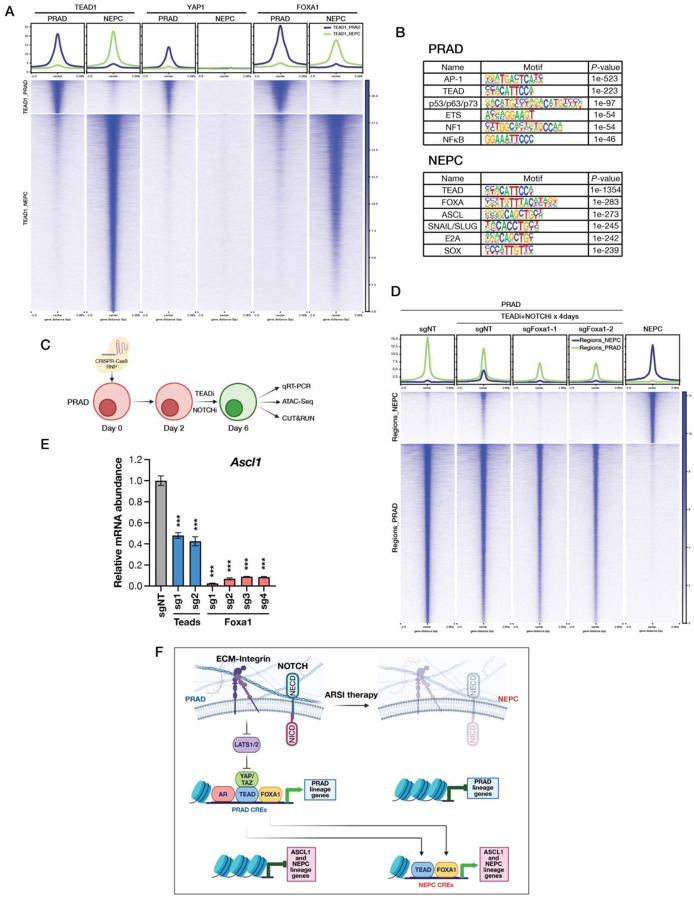
FOXA1 is essential for the PRAD to NEPC lineage transition. (A) CUT&RUN profiles of TEAD1, YAP1 and FOXA1 in TKO PRAD and NEPC organoids. Clusters were defined based on differential TEAD1 binding between PRAD and NEPC. (B) Top enriched transcription factor binding motifs identified by HOMER known-motif analysis of TEAD1 CUT&RUN peaks in TKO PRAD and NEPC organoids. (C) Schematic illustrating the experimental design used to assess the role of Teads and Foxa1 in driving the PRAD-to-NEPC transition. (D) ATAC-seq peak heatmaps showing chromatin accessibility across PRAD and NEPC-specific regions across the indicated organoid lines and conditions. Differential peaks were identified following depth normalization of ATAC-seq signal. (E) qRT-PCR for *Ascl1* in TKO PRAD organoids treated with 1uM IAG933 and 10uM DAPT following knockout of the indicated TFs. Error bars, ±SEM, n=3, ***p<0.001. (F) Model showing how YAP/TEAD, NOTCH and AR regulate the PRAD to NEPC lineage transition. ECM–integrin signaling activates YAP/TEAD by LATS1/2 inhibition to maintain PRAD lineage identity, in cooperation with NOTCH and AR. Combined inhibition of YAP/TEAD, NOTCH, and AR pathways reprograms PRAD cells into NEPC lineage. Early in this transition, FOXA1 and TEAD relocalize from PRAD cis-regulatory elements (CREs) to NEPC CREs, which leads to opening of new chromatin, activation of *ASCL1* expression and commitment to NE lineage.

To explore the specific molecular events responsible for the PRAD to NEPC lineage transition, we initially focused on the YAP/TEAD axis, using CUT&RUN to map the chromatin binding sites of YAP1 and TEAD1 across the PRAD and NEPC lineages. Focusing initially on TEAD1 peaks, we observed a remarkable re-localization of binding sites when comparing the PRAD state to the NEPC state (Fig. 6A). In PRAD, genes and pathways enriched at the sites of TEAD peaks include actin filament organization, epithelial morphogenesis, and cell substrate adhesion, all consistent with an adenocarcinoma lineage (exemplified by *Tacstd2*). Conversely, genes and pathways enriched at the sites of the redistributed TEAD peaks in the NEPC state include regulation of neurogenesis, forebrain development and synapse pathways (exemplified by *Ascl1*) (Fig. S6A–D).

YAP1 peaks in the PRAD state overlapped precisely with TEAD1 peaks, as expected, since these two proteins form a complex but were absent in the NEPC state because LATS kinase activation disrupts the YAP/TEAD complex (Fig. 6A). The fact that robust TEAD peaks are now detected at the enhancers of NEPC-specific genes in the absence of YAP raises the question whether TEAD binding plays a role in their expression. To address this, we performed CRISPR experiments targeting TEAD1, the most abundantly expressed of the four TEAD family members in the NEPC state and achieved robust knockdown of TEAD1 expression, albeit with some residual TEAD expression detected using a pan TEAD antibody, presumably from other TEAD family members (Fig. S6E). Despite this caveat, *Ascl1*, *Chga* and *Ncam1* mRNA and protein were reduced by ~50% following TEAD1 knockdown (Fig S6F), evidence that the re-localized TEAD peaks play a role in maintenance of NE-specific gene expression despite the absence of YAP and TAZ (Fig 5F). Because TEADs are DNA binding proteins without intrinsic coactivation function, it will be of interest to determine whether another (perhaps NE-specific) coactivator is recruited to perform this function.

To further investigate the biology underlying the NEPC-specific TEAD1 peaks, we performed motif analysis. TEAD binding motifs were enriched in both PRAD and NEPC lineages, but there were clear lineage-specific differences in co-enriched motifs, such as FOXA and ASCL1 binding sites in NEPC (Fig. 6B). The fact that FOXA motifs are co-enriched at the NEPC-specific TEAD binding sites raises the possibility that FOXA1, which is abundantly expressed in both PRAD and NEPC states, may play a role in the lineage transition. To explore this possibility, we first performed CUT&RUN and confirmed that FOXA1 peaks are indeed present at the NEPC-specific TEAD binding sites and, like TEAD, re-localize from PRAD to NEPC specific enhancers (Fig. 6A). Because FOXA1 is a pioneer factor, we postulated that FOXA1 mobilization might be an early step in the NEPC transition, catalyzing the opening of closed chromatin at NEPC specific enhancers regions and thereby enabling binding of NEPC lineage TFs. To test this hypothesis, we treated the PRAD cells with DMSO versus TEADi + NOTCHi (+/− CRISPR deletion of *Foxa1*) to assess changes in open chromatin and to determine whether FOXA1 is required for the NE transition (Fig. 6C). ATAC-seq experiments revealed a global increase in open chromatin at NE-specific loci after 4 days that was diminished in FOXA1-deleted cells (Fig. 6D), consistent with the known role of FOXA1 as a pioneer factor. FOXA1 deletion also completely abrogated *Ascl1* induction (Fig. 6E), consistent with the absence of FOXA1 peaks at the *Ascl1* enhancer based on CUT&RUN experiments (Fig. S6G). TEAD knockdown lowered the magnitude of *Ascl1* induction by ~50% (Fig. 6E, S6H), similar to the effect in established NEPC organoids (Fig S6F). Together, these findings establish that FOXA1, likely through its role as a pioneer factor, is a key upstream regulator of *Ascl1* induction during the PRAD to NEPC transition.

## Discussion

Next generation ARPIs extend prostate cancer survival through increased selective pressure on AR. Despite this clinical success, tumor cells can escape AR dependence by transitioning from AR-dependent luminal cells to AR-independent NEPC cells (lineage plasticity). Here we show that the YAP/TEAD pathway plays a key role in maintenance of the PRAD lineage state, preventing tumor cells from undergoing the PRAD to NEPC lineage switch. Prior work on HIPPO pathway signaling, initially in flies and subsequently in mammalian cells, has established a critical role of YAP/TEAD in growth regulation (29, 38–40). In the context of tumor cells, the role of YAP/TEAD in growth regulation is more nuanced. Studies with pharmacologic inhibitors reveal YAP/TEAD dependency in cancers with HIPPO pathway mutations (such as mesotheliomas with NF2 loss) but not more broadly, even in tumors with robust YAP/TEAD activation (26, 41). Here we provide evidence here that YAP/TEAD functions as a gatekeeper, preventing prostate epithelial tumor cells at risk for lineage plasticity from undergoing a lineage transition.

Using PRAD as a model, we find ECM engagement is the primary mechanism of YAP/TEAD activation, consistent with prior evidence linking YAP/TEAD to integrins and mechanical stress (25). Once PRAD tumor cells lose contact with ECM (by experimental withdrawal in organoid culture or stochastically during *in vivo* tumor expansion), YAP/TEAD activity is acutely switched off, followed by induction of ASCL1 and an eventual transition to NEPC. Importantly, this switch cannot be easily reversed by reengagement with ECM because integrin expression (which itself is YAP/TEAD dependent) is lost. Thus, tumor cells poised for lineage transition (i.e., those with RB loss) enter a point of no return once the YAP/TEAD signal is switched off. Of note, YAP/TEAD has also been implicated in other lineage transitions such as reversion to a fetal-like state in a colorectal cancer model (42) and an epithelial mesenchymal-like state in a lung cancer model (43). Importantly, in these examples YAP/TEAD activity is oncogene-driven and promotes plasticity. In contrast, our work shows the ECM/integrin-driven YAP/TEAD activation restrains plasticity in PRAD cells primed for lineage transition due to *RB1* and *TP53* loss.

Interestingly, NE lineage transition following loss of ECM-integrin engagement occurs in a mosaic pattern, suggesting cell-cell interactions provide an additional brake on the PRAD to NEPC lineage independent of YAP/TEAD. Recognizing the critical role of NOTCH in cell-cell interactions, we explored NOTCH signaling in our organoid model through gamma secretase inhibition and found remarkable cooperativity of NOTCH and YAP/TEAD in maintenance of the PRAD state, consistent with evidence that NOTCH can suppress NE lineage transitions in prostate and lung cancer models (34, 35).

We also find that ARPI therapy, the therapeutic intervention that initiates the NEPC transition in CRPC patients, contributes to the PRAD to NEPC transition, particularly when YAP/TEAD or NOTCH signaling (or both) have already been impaired. The mechanism is cell autonomous because the NE lineage transition can be fully elicited in organoid cultures that only contain tumor cells. A plausible explanation is that AR plays a role in maintenance of luminal lineage identity, even in CRPC. That said, non-cell autonomous mechanisms may also be at play. Intriguingly, ECM production by stromal cells (e.g., collagen) in the normal mouse prostate is AR-regulated (44). One consequence of systemic ARPI therapy could be reduced YAP/TEAD activation in tumor cells due to reduced ECM production by AR-positive stromal cells, thereby lowering the threshold for NEPC transition to occur.

Our ability to fully recapitulate the PRAD to NEPC transition *in vitro* provided an opportunity to investigate the molecular details of this dynamic process at a level of resolution and efficiency not easily achievable *in vivo*. Toward that end, we first examined TEAD chromatin binding of across the genome and uncovered a striking redistribution of TEAD peaks from PRAD-specific to NEPC-specific enhancers. Equally striking was a parallel redistribution of FOXA1 peaks, raising the intriguing possibility that FOXA1, a pioneer factor expressed in both PRAD and NEPC, plays a pivotal role in initiating the lineage transition. Indeed, CRISPR deletion studies establish that FOXA1 is essential for the reshaping of the open chromatin landscape triggered by TEAD + NOTCH inhibition in PRAD organoids (see model, Fig. 6F).

Despite this new clarity, several questions remain to be addressed. While there is abundant evidence that RB loss lowers the threshold to undergo plasticity, the mechanism is unclear. A recent ChIP-seq study (performed in retinal pigment epithelial (RPE1) cells) revealed RB binding at enhancers, distinct from canonical RB binding sites in the promoters of genes that regulate cell cycle progression. In contrast to E2F motifs that are enriched at promoter binding sites (where RB is a transcriptional repressor), RB binding sites at enhancers are enriched for TEAD and AP1 motifs (where RB now functions as a coactivator) (45). The fact that AP1 motifs are similarly enriched at TEAD binding sites in PRAD lineage organoids (Fig. 6B) provides a remarkable parallel to the RPE1 results, raising the intriguing possibility that RB binding at these sites locks cells into an epithelial lineage state. A plausible model is that once RB loss occurs, maintenance of an epithelial lineage state becomes more tenuous, with sustained YAP/TEAD activity playing a critical role together with input from NOTCH and AR (Fig. 6F).

The fact that lineage plasticity in our models can be reversed by restoring YAP/TEAD activity through LATS kinase inhibition (or genetically through constitutive YAP activation) raises the intriguing possibility that CRPC patients at risk for plasticity could benefit from treatment with a clinical grade LATS inhibitor. Such an intervention might not only reinforce PRAD lineage maintenance but also reprogram tumors with mixed lineage phenotypes (an increasingly common clinical phenotype) to a homogeneous PRAD state. Because sustained systemic LATS inhibition is likely to be poorly tolerated (based on hyperproliferation syndromes seen in LATS deficient mice) (46), evaluation of this “lineage restoration” approach in CRPC would likely require transient LATS inhibition followed by treatment with definitive PRAD therapy. Novel ARPIs might be one option (e.g., AR degraders), but emerging treatments using radioligands, antibody drug conjugates or T cell engagers directed at PRAD-specific cell surface antigens (such as PSMA or KLK2) may be more promising. It will also be important to explore whether the importance of YAP/TEAD activation in PRAD lineage maintenance reported here extends to other tumor settings in which lineage plasticity is also a mechanism of acquired resistance, such as EGFR-mutant lung cancer.

## Supplementary Material

1

## Data Availability

Uploading of sequencing data to the Gene Expression Omnibus (GEO) database is pending due to the current government shutdown. All other data is available in the manuscript or the supplementary materials.
