# Supplementary material for: Extracellular matrix regulates lineage plasticity in prostate cancer through YAP/TEAD
Source: bioRxiv. 2025 Dec 31:2025.12.30.697072. Preprint. [Version 1] doi: 10.64898/2025.12.30.697072 (PMC12776418; doi:10.64898/2025.12.30.697072)
Supplement: 1 [file NIHPP2025.12.30.697072V1-supplement-1.pdf]

bioRxiv preprint doi: https://doi.org/10.64898/2025.12.30.697072; this version posted December 31, 2025. The copyright holder for this preprint (which was not certified by peer review) is the author/funder, who has granted bioRxiv a license to display the preprint in perpetuity. It is made available under aCC-BY 4.0 International license.

Supplementary Materials:

Materials and Methods

Ethical statement

Mouse experiments were conducted under protocol 0607012 approved by the Institutional Animal Care and Use Committee of Memorial Sloan Kettering Cancer Center (MSKCC), New York.

Organoid culture, analysis and transplantations

Mouse prostate organoid derivation and culture

Whole mouse prostates, including all lobes, were isolated as previously described (*47*, *48*). Briefly, dissected prostates were digested with collagenase type II (Gibco) for 2 hours at 37 °C, followed by TrypLE™ Express (Gibco) digestion at 37 °C until a single-cell suspension was obtained. All digestions were supplemented with Y-27632 (10 μM; MedChemExpress, HY-10583) to prevent anoikis, and the resulting cell suspension was filtered through 40-μm strainers to remove debris. Murine prostate organoids were established and maintained under standard culture conditions as previously described (*47*, *48*). In brief, dissociated epithelial cells were embedded in 20-μL drops of growth factor–reduced Matrigel (Corning, 356231) and overlaid with mouse prostate organoid medium.

Organoid genetic engineering

Genetic perturbation in organoids was performed using CRISPR–Cas9 ribonucleoprotein (RNP) complexes as previously described (*49*). Briefly, Cas9 protein (IDT) was incubated with synthetic sgRNA (IDT) to assemble the RNP complex prior to nucleofection. A total of 1.2 μM cRNP was used per sgRNA. Dissociated organoid cells (5×10^5–1×10^6) were resuspended in nucleofection buffer containing RNP complexes and electroporation enhancer (IDT; 1:1 molar ratio to RNP) in a final volume of 100 μL. The suspension was transferred to a nucleofection cuvette and electroporated using a Lonza Amaxa Nucleofector II (program T-030). Cells were then centrifuged and seeded into Matrigel for culture. Pten-null organoids were enriched by withdrawal of EGF from the culture medium.

bioRxiv preprint doi: https://doi.org/10.64898/2025.12.30.697072; this version posted December 31, 2025. The copyright holder for this preprint (which was not certified by peer review) is the author/funder, who has granted bioRxiv a license to display the preprint in perpetuity. It is made available under aCC-BY 4.0 International license.

For lentiviral transduction, viral titers were determined prior to infection, and a multiplicity of infection (MOI) of 0.2 was used to ensure single-copy integration. A total of 5 × 10^5 cells were used per reaction. cMyc overexpression was achieved by lentiviral transduction of a construct containing *cMyc–IRES–eGFP*; GFP-positive cells were isolated by fluorescence-activated cell sorting (FACS).

For adenoviral transduction, 1 µL of Ad-CMV-Null (Vector Biolabs, 1300) or 1 µL of Ad-CMV-iCre (Vector Biolabs, 1045) was used to infect 5 × 10^4 cells. To enhance infection efficiency, spinoculation was performed at 32 °C and 600 × *g* for 1 hour.

RNA isolation, cDNA Synthesis and quantitative PCR

RNA was isolated from organoids using the RNeasy Plus Mini Kit (Qiagen) according to manufacturer's protocol. RNA concentration was quantified using a NanoDrop (ThermoFisher). Complementary DNA (cDNA) was synthesized using High-Capacity cDNA Reverse Transcription Kit (Thermo Scientific) according to manufacturer's instructions. Quantitative PCR experiments were conducted on Applied Biosystems QuantStudio 6 Flex Real-Time PCR system. qPCR primers were listed in Supplementary Table.

Suspension culture

Organoid cells were dissociated using TrypLE™ Express Enzyme (Gibco) and passed through 40um cell strainers (Fisher Scientific). A total of 1 x 10^6 cells were resuspended in prostate organoid medium and seeded into one well of a Nunclon™ Sphera™ plate (Thermo Scientific, 174932) or an Ultra-low attachment plate (Corning, 3471). Cells were passaged every 3-4 days. For passaging, cells were enzymatically dissociated to single cells using TrypLE™ Express prior to reseeding.

Protein isolation and western blot analysis

Organoids were isolated from the Matrigel using cell recovery solution (Corning, 354253). Cells were lysed in RIPA buffer containing protease inhibitors (Calbiochem) and phosphatase inhibitors (Calbiochem). Protein concentrations were quantified using a bicinchoninic acid (BCA) assay

bioRxiv preprint doi: https://doi.org/10.64898/2025.12.30.697072; this version posted December 31, 2025. The copyright holder for this preprint (which was not certified by peer review) is the author/funder, who has granted bioRxiv a license to display the preprint in perpetuity. It is made available under aCC-BY 4.0 International license.

(Pierce, Thermo Fisher). Lysates were denatured using 4X protein loading dye (SDS 200 nM Tris, 8% SDS, 0.4% bromophenol blue, 40% glycerol, 400 mM 2-mercaptoethanol, pH 6.8). 20-30 µg of protein was loaded on NuPage 4-12% gradient polyacrylamide gels (Invitrogen). After electrophoresis, protein was transferred to a PVDF membrane and blocked with 5% milk in TBS-T. Primary antibodies were incubated overnight at 4 degrees. Membranes were washed using TBS-T and incubated with secondary antibodies for 1 h at room temperature with shaking. Proteins were visualized using ECL prime (Amersham, GE healthcare) and ImageQuant 800 (Amersham, GE healthcare). Antibodies used in this study are listed in the Supplementary Table.

Orthotopic transplantation

Organoid cells (2 x 10^5) were resuspended in 20 µl of a 1:1 mixture of growth factor-reduced Matrigel (Corning, 356231) and organoid culture medium and injected into the prostate dorsal lobes of immunodeficient NSG mice (JAX 005557) or C57BL/6J mice (JAX 000664) at 2 months of age.

Subcutaneous transplantation

Organoid cells (2 x 10^5) were resuspended in 100 µl of a 1:1 mixture of growth factor-reduced Matrigel (Corning, 356231) and organoid culture medium and injected subcutaneously into the right flank of immunodeficient NSG mice (JAX 005557) or C57BL/6J mice (JAX 000664) at 2 months of age.

Generation of NEPC tumoroids

Mouse prostate tumors were dissected and finely minced using sterile surgical blades. Tumor fragments were enzymatically dissociated in collagenase type II (Gibco) for 1 hour at 37 °C, followed by further digestion with TrypLE™ Express (Gibco) for 30 minutes at 37 °C. All digestions were supplemented with Y-27632 (10 µM) to prevent anoikis. The resulting cell suspension was passed through 40-µm cell strainers (Fisher Scientific) to obtain a single-cell suspension. Cells were stained on ice for 1 hour with CD51-PE (1:200; BioLegend, 104106) and EpCAM-Alexa

bioRxiv preprint doi: https://doi.org/10.64898/2025.12.30.697072; this version posted December 31, 2025. The copyright holder for this preprint (which was not certified by peer review) is the author/funder, who has granted bioRxiv a license to display the preprint in perpetuity. It is made available under aCC-BY 4.0 International license.

Fluor 647 (1:200; Abcam, ab237385). $CD51^-$/$EpCAM^+$ cells were isolated by fluorescence-activated cell sorting (FACS) and cultured under standard prostate organoid culture conditions.

Histology and immunostaining

For GEMM experiments, whole mouse prostates containing all lobes were collected at autopsy. Histological data from all lobes were pooled for quantification. For intraprostatic transplantation and adenoviral infection experiments, injected prostate lobes were collected at autopsy. Prostate tissues were fixed using 4% paraformaldehyde, dehydrated with 70% ethanol, paraffin-embedded and sectioned. H&E staining was performed following standard protocols by the MSKCC Molecular Cytology Core. Immunohistochemistry and immunofluorescence were performed on a Leica Bond RX automatic stainer using antibodies listed in the Supplementary Table. Formalin-fixed, paraffin-embedded (FFPE) stained tissue sections were scanned using a Pannoramic P250 Flash scanner (3DHISTECH, Hungary) equipped with a 20×/0.8 NA objective. Whole-slide images were exported as .tif files using *SlideViewer* software (3DHISTECH, Hungary) for subsequent analysis in *ImageJ/FIJI* (NIH, USA). For immunohistochemistry (IHC) quantification, color deconvolution was applied to separate hematoxylin and DAB signals. The tissue area and DAB-positive area were determined by thresholding, and the ratio of DAB-positive area to total tissue area was used as a quantitative measure of staining intensity. For immunofluorescence (IF) quantification, multi-channel images were similarly exported and analyzed in *ImageJ/FIJI*. Tissue area was defined using the DAPI channel. Nuclei were segmented by generating a DAPI mask followed by watershed separation. Regions of interest (ROIs) approximating individual cells were then used to assess marker positivity, defined by the percent area of signal per marker channel relative to the nuclear ROI.

Bulk RNA-seq

RNA was extracted using the RNeasy Plus Mini Kit (Qiagen) from bulk samples and sequenced at the Integrated Genomics Operation Core (MSKCC). cDNA was synthesized from purified RNA using oligo(dT) primers and reverse transcriptase according to standard Illumina protocols. The

bioRxiv preprint doi: https://doi.org/10.64898/2025.12.30.697072; this version posted December 31, 2025. The copyright holder for this preprint (which was not certified by peer review) is the author/funder, who has granted bioRxiv a license to display the preprint in perpetuity. It is made available under aCC-BY 4.0 International license.

resulting cDNA was subjected to automated Illumina paired-end library construction. Libraries were sequenced on Illumina HiSeq2000 instruments with paired reads of 100 base pairs (bp) in length per sample, generating approximately 30–40 million reads per sample. Sequence data were processed and analyzed using Partek^{TM} Flow^{TM} software, v11.0. Briefly, raw FASTQ files were quality-checked and trimmed to remove low-quality bases and adaptor sequences. Cleaned reads were aligned to the mouse reference genome (mm10) using the STAR aligner with default parameters. Aligned reads were quantified at the gene level based on Ensembl transcript annotations. Gene-level read counts were normalized using the Fragments Per Kilobase of transcript per Million mapped reads (FPKM) method to account for sequencing depth and transcript length. For visualization, normalized expression values were log2-transformed. Heatmaps and principal component analysis (PCA) plots were generated using normalized expression values to visualize global transcriptomic differences among samples. Differential gene expression was analyzed using the DESeq2 algorithm (v1.34.0). Genes with an adjusted $p < 0.05$ and absolute log2 fold change > 2 were considered significantly differentially expressed. Gene set enrichment analysis (GSEA) was performed using the GSEA software (https://www.gsea-msigdb.org/gsea/index.jsp)

Bulk RNA-seq analysis on patient cohort

RNA-seq data from two clinical cohorts were obtained from the cBioPortal for Cancer Genomics (*50*). The cohorts included: the SU2C/PCF Dream Team cohort (210 adenocarcinoma and 22 neuroendocrine prostate cancer samples) (*12*), and the Beltran *et al.* cohort (*23*).

YAP/TAZ target score and Integrin score were calculated using Gene Set Variation Analysis (GSVA) with the single-sample Gene Set Enrichment Analysis (ssGSEA) method (*51*), implemented in R (version 4.3.2). The YAP/TAZ target gene set was derived from Wang et al (*52*), and the Integrin score was calculated using all annotated integrin genes as the input gene set. Statistical differences in gene set scores and mRNA expression levels of individual genes

bioRxiv preprint doi: https://doi.org/10.64898/2025.12.30.697072; this version posted December 31, 2025. The copyright holder for this preprint (which was not certified by peer review) is the author/funder, who has granted bioRxiv a license to display the preprint in perpetuity. It is made available under aCC-BY 4.0 International license.

between adenocarcinoma and NEPC samples were assessed using a two-tailed Wilcoxon rank-sum test.

Visium spatial transcriptomics

Spatial transcriptomics acquisition and preprocessing

Visium spatial transcriptomics data were downloaded from GEO accession GSE278936, including spatial alignments, high-resolution histology images, and spot-level gene expression matrices. After loading the data into Scanpy, low-quality spots (<500 total UMIs) and genes expressed in fewer than three spots were filtered on a per-sample basis. Gene counts were deduplicated by summing counts for features sharing the same gene symbol.

Single-cell reference atlas

Single-cell RNA sequencing data comprising 119,083 cells from prostate tumors were obtained from Zaidi et al. (2024) (28) ("Single-cell analysis of treatment-resistant prostate cancer") as downloaded from GEO (GSE264573). This dataset includes annotations for major cell types, including myeloid, lymphoid, stromal, normal epithelial, and tumor epithelial cells. Tumor epithelial cells in this dataset are further subclassified into castration-sensitive prostate cancer (CSPC), castration-resistant prostate cancer (CRPC), and neuroendocrine prostate cancer (NEPC).

Cell type deconvolution using BayesPrism

Because Visium spots capture transcripts from multiple cells, spot-level deconvolution was performed to infer underlying cell type composition in the Kiviaho et al. (2024) (18) spatial dataset. Reference-based spatial deconvolution was performed using BayesPrism.

To improve deconvolution accuracy, a feature selection step was performed following recommendations from the BayesPrism authors. Marker genes were identified from the Zaidi et al. (2024) (28) single-cell reference by performing pairwise differential gene expression analysis between cell types using Scanpy's rank_genes_groups function with the "t-test_overestim_var" method applied to log-normalized gene expression values. Genes were retained if they exhibited

bioRxiv preprint doi: https://doi.org/10.64898/2025.12.30.697072; this version posted December 31, 2025. The copyright holder for this preprint (which was not certified by peer review) is the author/funder, who has granted bioRxiv a license to display the preprint in perpetuity. It is made available under aCC-BY 4.0 International license.

a log fold change ≥ 0.25 and an adjusted p-value < 0.05 across all relevant pairwise comparisons. This feature selection strategy was chosen to reduce noise from non-informative genes and improve deconvolution stability.

The number of retained marker genes per cell type or state was as follows: CSPC (1,846), NEPC (7,786), CRPC (3,314), non-tumor epithelial (1,336), lymphoid (1,405), myeloid (2,195), and stromal (2,334).

Spot-level raw gene counts from each Visium sample and cell-level raw counts from the single-cell reference atlas were used as inputs to BayesPrism (pybayesprism). For each sample, expression matrices were restricted to genes shared between the spatial data and the selected marker gene lists.

BayesPrism yields fractional contribution (“theta”) estimates for each cell state per spot, which were used in all downstream analyses.

BayesPrism deconvolution robustness analysis via downsampling

To assess the robustness of BayesPrism deconvolution given the sparsity of Visium data, we performed UMI downsampling analyses. For each of the 48 Visium slides, BayesPrism deconvolution was repeated 20 times following random downsampling of 10% of UMIs per slide. Pairwise Spearman correlations (190 comparisons per sample) were computed between theta estimates derived from downsampled runs for each cell type. Spearman correlation was chosen due to the non-normal distribution of theta estimates.

Pathologist annotation–based spot-level validation

To validate deconvolution results, spot-level pathological annotations provided by the authors of Kiviaho et al. (2024) (18) were compared with BayesPrism estimates. Pathologist annotation categories included Stroma, Benign, Inflammation, Atrophy, PIN, and tumor-associated spots annotated by Gleason score (e.g., Gleason X). Notably, prostate tumor subclasses such as NEPC or neuroendocrine-like pathology were not included in the pathological annotations and therefore could not be independently assessed.

bioRxiv preprint doi: https://doi.org/10.64898/2025.12.30.697072; this version posted December 31, 2025. The copyright holder for this preprint (which was not certified by peer review) is the author/funder, who has granted bioRxiv a license to display the preprint in perpetuity. It is made available under aCC-BY 4.0 International license.

Of 112,080 spots retained following quality control and filtering, 25,186 spots had corresponding pathologist annotations. For these spots, the total deconvolved tumor fraction (defined as the sum of CSPC, CRPC, and NEPC theta estimates) and the deconvolved fibroblast fraction were compared with pathological annotations.

Analysis of spatial co-localization of tumor and stroma

A spot was classified as NEPC-high, CRPC-high, CSPC-high, or stroma-high if its corresponding theta estimate exceeded 0.25. Spots exceeding this threshold for both a tumor cell state and stromal content were classified as mixed. To assess sensitivity to threshold choice, tumor theta thresholds were varied from 0.1 to 0.4, and downstream analyses were repeated.

Single-cell multiome

Data pre-processing

The FASTQ files of the single-cell multiome data were processed by sample with cellranger-arc v. 2.0.2 with alignment against reference genome mm10. All samples were aggregated using the cellranger-arc aggr function v. 2.0.2. The resulting consensus peak list of genomic regions was annotated with the HOMER (*53*) function version 4.11 with the cellranger-arc refdata-cellranger-arc-mm10-2020-A-2.0.0 genes.gtf file.

Quality control

Cell filtering criteria were calculated independently for each sample and modality. For single-cell gene expression data, we determined the library size per cell, the number of genes expressed per cell, and the fraction of mitochondrial counts per cell. Specifically, cells expressing fewer than 1,000 or more than 10,000 genes, those with fewer than 1,000 or more than 20,000 total UMI counts, or those exhibiting a mitochondrial read fraction exceeding 20% were excluded. Following this initial cellular filtration, a doublet detection score was computed using Scrublet (*54*) v. 0.2.3, employing the parameters min_counts = 2, min_cells = 3, vscore_percentile = 85, n_pc = 50, expected_doublet_rate = 0.02, sim_doublet_ratio = 3, and n_neighbors = 15. Subsequently, all

bioRxiv preprint doi: https://doi.org/10.64898/2025.12.30.697072; this version posted December 31, 2025. The copyright holder for this preprint (which was not certified by peer review) is the author/funder, who has granted bioRxiv a license to display the preprint in perpetuity. It is made available under aCC-BY 4.0 International license.

cells with a doublet score greater than 0.14 were removed. Finally, genes expressed in fewer than 3 cells were discarded.

For single-cell chromatin accessibility data, we assessed the minimum library size per cell, the number of peaks detected per cell, the maximum TSS enrichment score, and the nucleosome signal (defined as the ratio of nucleosome-free to mono-nucleosome fragments per cell). Specifically, cells with fewer than 500 or more than 20,000 peaks, or with a total UMI count below 1,000 or above 40,000, were excluded. Additionally, all peaks detected in fewer than 50 cells were removed. Cells were filtered separately for gene expression and chromatin accessibility, with only those cells passing quality control in both modalities being retained. The resulting dataset comprised 23,134 cells, 23,312 genes, and 147,895 peaks.

Normalization and feature selection

For single-cell GEX, normalization was performed using a shifted log-transformation of counts, divided by the library size and scaled to 10,000 reads (logCPM+1). For single-cell ATAC normalization, Term Frequency - Inverse Document Frequency (TF-IDF) was computed to weigh the importance of open chromatin regions. Specifically, a shifted log-transformation of the term frequency (TF) was utilized, a method demonstrated to be beneficial in sparse datasets compared to direct TF application, as implemented in the muon (*55*) ATAC module.

To select highly variable genes, the scanpy (*56*) (v. 1.10.3) function pp.compute_highly_variable was employed with the ‘cellranger’ flavor, resulting in the selection of 3,000 highly variable genes per sample. Highly variable peaks were computed using the scanpy function pp.compute_highly_variable, with parameters min_mean=0.05 and min_disp=0.5, yielding 16,865 highly variable peaks.

Cell cycle genes were scored to determine the cell cycle phase using the scanpy tl.score_genes_cell_cycle function per sample. As a reference, the human cell cycle gene list curated by Tirosh et al. (*57*) was converted to align with the mouse gene symbol convention.

Dimensionality reduction

bioRxiv preprint doi: https://doi.org/10.64898/2025.12.30.697072; this version posted December 31, 2025. The copyright holder for this preprint (which was not certified by peer review) is the author/funder, who has granted bioRxiv a license to display the preprint in perpetuity. It is made available under aCC-BY 4.0 International license.

For dimensionality reduction, principal component analysis (PCA) was applied to gene expression data, and latent semantic indexing (LSI) (*58*) was utilized for chromatin accessibility. A weighted nearest neighbor (WNN) graph, incorporating L2-regularization of distances derived from both modalities on highly variable features, was then computed to generate a joint UMAP.

Batch correction

We did not perform any batch correction in the final dataset, because all samples were processed as a single batch.

Clustering and annotation

Leiden (*59*) clustering, at a resolution of 0.5, resulted in seven distinct clusters. Gene characterization was subsequently performed utilizing a t-test within the scanpy tl.rank_genes_groups function, employing a one-versus-rest comparison. Differentially accessible peaks were characterized using the Wilcoxon rank-sum test, also within the scanpy tl.rank_genes_groups function and a cluster-versus-rest setup. The top 20 genes/peaks were then investigated to characterize each cluster. The smallest cluster, comprising 444 cells from all three samples, was subsequently excluded.

All analyses were performed in Python v. 3.10.15.

Chromatin profiling and data analysis

Bulk ATAC-seq

Freshly harvested prostate organoid cells were processed by MSKCC's Epigenetics Research Innovation Lab. ATAC was performed using 50,000 cells per replicate as previously described (*60*) using the OpenTn5 enzyme (*61*). The sequencing libraries were purified with SPRIselect magnetic beads (B23318, Beckman Coulter), quantified using a Qubit Flex fluorometer (ThermoFisher Scientific) and profiled using a TapeStation 4200 (Agilent). The libraries were sequenced by the MSKCC Integrated Genomics Operation core facility. After PicoGreen quantification and quality control by Agilent TapeStation, libraries were pooled and run on a NovaSeq 6000 in a PE100 run, using the NovaSeq 6000 S4 Reagent Kit (200 Cycles) (Illumina).

bioRxiv preprint doi: https://doi.org/10.64898/2025.12.30.697072; this version posted December 31, 2025. The copyright holder for this preprint (which was not certified by peer review) is the author/funder, who has granted bioRxiv a license to display the preprint in perpetuity. It is made available under aCC-BY 4.0 International license.

The loading concentration was 0.5nM and a 1% spike-in of PhiX was added to the run and for quality control purposes. The run yielded on average 50-60M reads per sample.

CUT&RUN

Freshly harvested prostate organoid cells were processed by MSKCC's Epigenetics Research Innovation Lab. CUT&RUN was performed with 1M cells per replicate using the CUTANA™ ChIC/CUT&RUN Kit (Epicypher #14-1048) and the following antibodies: rabbit polyclonal anti-FOXA1 (Invitrogen PA5-27157); rabbit monoclonal anti-YAP1 (CST, #14074); rabbit monoclonal anti-TEAD1 (CST, #12292); rabbit anti-H3K4me3 (Epicypher, #13-0041); rabbit anti-mouse IgG (Epicypher, #13-0042). The recovered DNA fragments were quantified and sent to the MSKCC Integrated Genomics Operation core facility for library preparation and sequencing. Immunoprecipitated DNA was quantified by PicoGreen and the size was evaluated by Agilent BioAnalyzer. Illumina sequencing libraries were prepared using the KAPA EvoPrep Kit (Roche 10212250702) according to the manufacturer's instructions with 0.2-5 ng input DNA and 14 cycles of PCR. Barcoded libraries were run on the NovaSeq 6000 in a PE100 run, using the NovaSeq 6000 S4 Reagent Kit (200 Cycles) (Illumina). An average of 5-10M paired reads were generated per sample.

Sequencing data analysis

Raw sequencing reads were trimmed and filtered for quality (Q>15) and adapter content using version 0.4.5 of TrimGalore (https://www.bioinformatics.babraham.ac.uk/projects/trim_galore) and running version 1.15 of cutadapt and version 0.11.5 of FastQC. Version 2.3.4.1 of bowtie2 (http://bowtie-bio.sourceforge.net/bowtie2/index.shtml) was employed to align reads to mouse assembly mm10 and alignments were deduplicated using MarkDuplicates in Picard Tools v2.16.0. Enriched regions were discovered using MACS2 (https://github.com/taoliu/MACS) with a p-value setting of 0.001, filtered for blacklisted regions (http://mitra.stanford.edu/kundaje/akundaje/release/blacklists/ mm10-mouse/mm10.blacklist.bed.gz), and a peak atlas was created using +/- 250 bp around peak

bioRxiv preprint doi: https://doi.org/10.64898/2025.12.30.697072; this version posted December 31, 2025. The copyright holder for this preprint (which was not certified by peer review) is the author/funder, who has granted bioRxiv a license to display the preprint in perpetuity. It is made available under aCC-BY 4.0 International license.

summits for ATAC data or using the entire ‘narrowPeak’ region for CUT&RUN data. The BEDTools suite (http://bedtools.readthedocs.io) was used to create normalized bigwig files. Version 1.6.1 of featureCounts (http://subread.sourceforge.net) was used to build a raw counts matrix and DESeq2 was employed to calculate differential enrichment for all pairwise contrasts for samples with replicates. For single sample data, MACS2 was run by swapping bams of different conditions to find differential regions. Clusters were discovered by creating a superset of all differential peaks then running k-means clustering from k=2:10 until cluster redundancy emerged. Peak-gene associations were created by assigning all intragenic peaks to that gene, while intergenic peaks were assigned using linear genomic distance to transcription start sites (TSS). Pathway enrichment was calculated by assigning each gene a unique score based on the associated peak with the greatest magnitude change and running GSEA in pre-ranked mode. Motif signatures were obtained using Homer v4.5 (http://homer.ucsd.edu) on differentially enriched peak regions. Composite and tornado plots were created using deepTools v3.3.0 by running computeMatrix and plotHeatmap on normalized bigwigs with average signal sampled in 25 bp windows and flanking region defined by the surrounding 2 kb. Network analysis was performed using enrichplot::cnetplot in R with default parameters.

bioRxiv preprint doi: https://doi.org/10.64898/2025.12.30.697072; this version posted December 31, 2025. The copyright holder for this preprint (which was not certified by peer review) is the author/funder, who has granted bioRxiv a license to display the preprint in perpetuity. It is made available under aCC-BY 4.0 International license.

Supplemental Figure 1. Cancer-associated fibroblasts and ECM are depleted in NEPC.

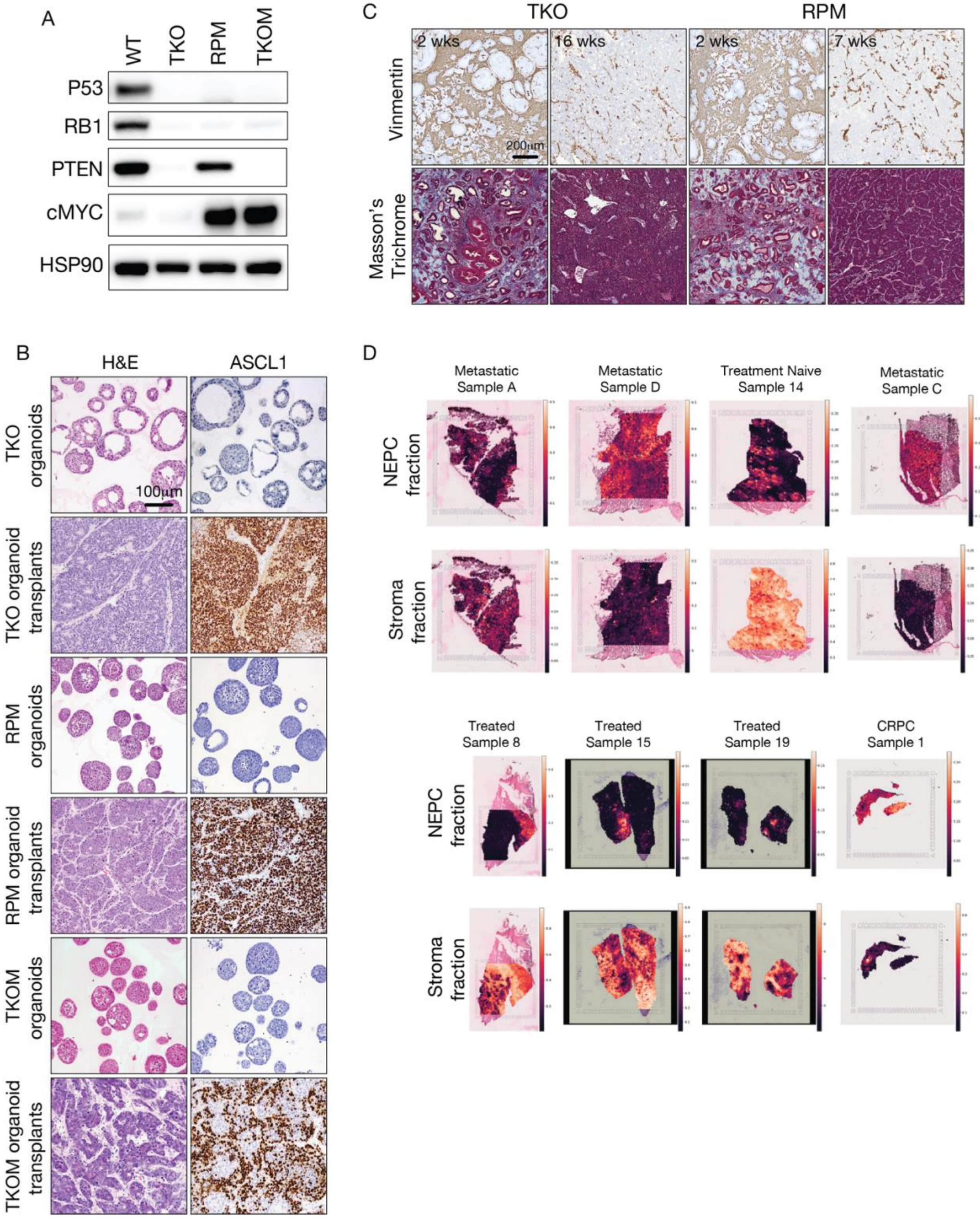

bioRxiv preprint doi: https://doi.org/10.64898/2025.12.30.697072; this version posted December 31, 2025. The copyright holder for this preprint (which was not certified by peer review) is the author/funder, who has granted bioRxiv a license to display the preprint in perpetuity. It is made available under aCC-BY 4.0 International license.

Supplemental Figure 2. Removal of extracellular matrix induces Ascl1 expression.

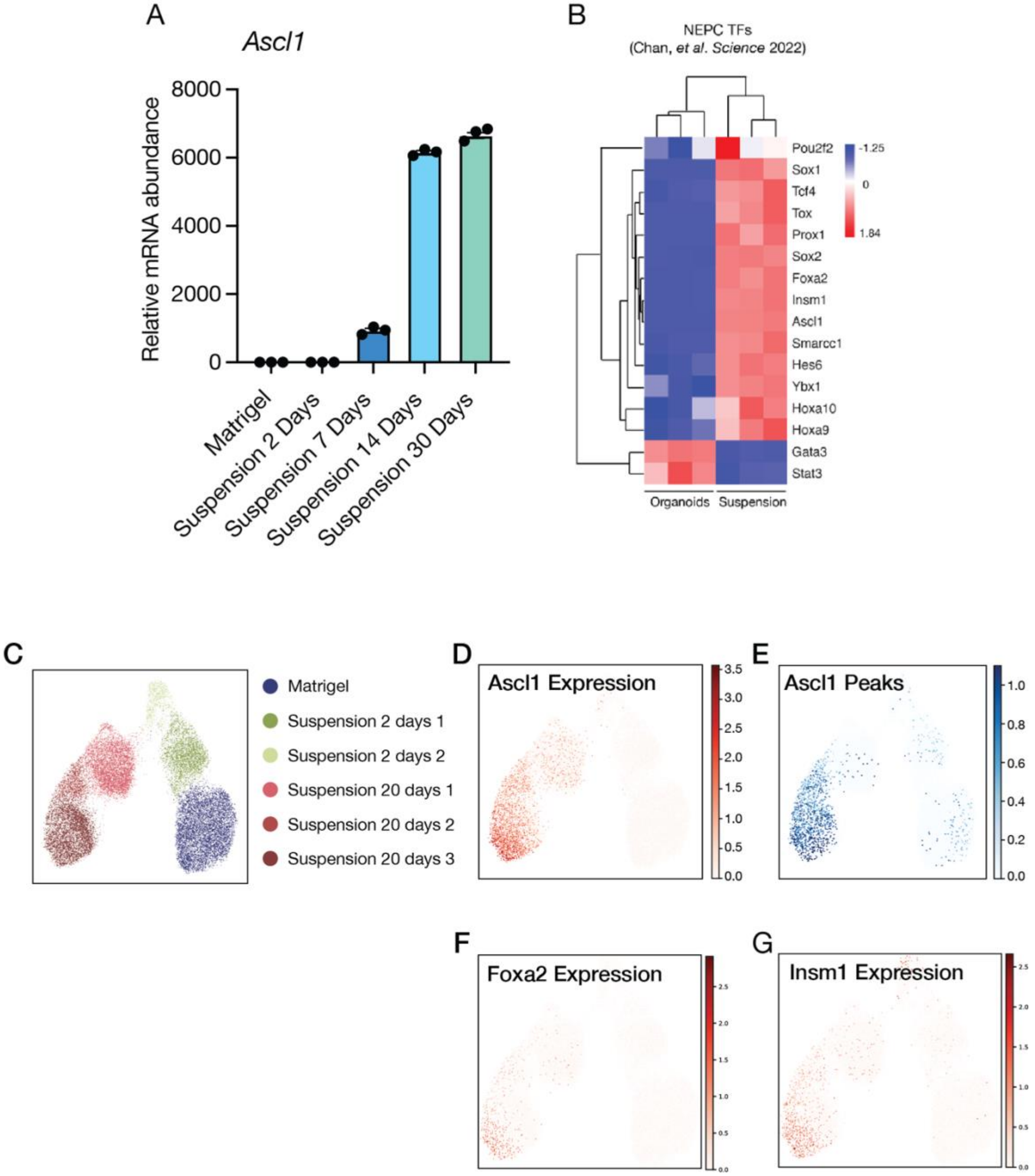

bioRxiv preprint doi: https://doi.org/10.64898/2025.12.30.697072; this version posted December 31, 2025. The copyright holder for this preprint (which was not certified by peer review) is the author/funder, who has granted bioRxiv a license to display the preprint in perpetuity. It is made available under aCC-BY 4.0 International license.

Supplemental Figure 3. YAP/TEAD activation blocks Ascl1 induction and PRAD to NEPC transition.

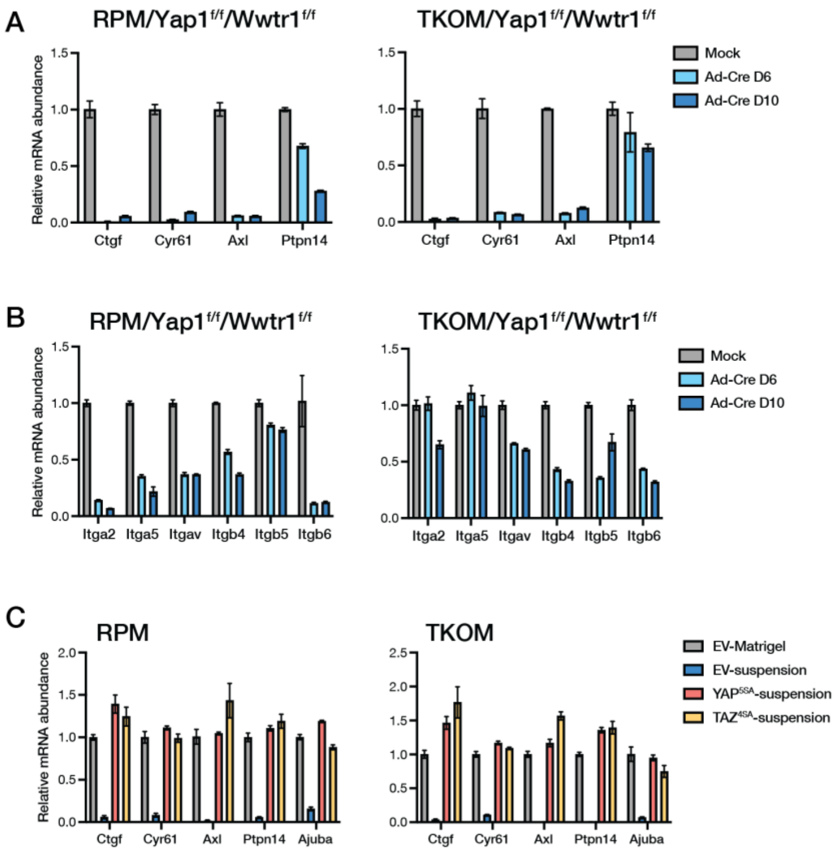

bioRxiv preprint doi: https://doi.org/10.64898/2025.12.30.697072; this version posted December 31, 2025. The copyright holder for this preprint (which was not certified by peer review) is the author/funder, who has granted bioRxiv a license to display the preprint in perpetuity. It is made available under aCC-BY 4.0 International license.

Supplemental Figure 4. LATS inhibition impairs acquisition and maintenance of neuroendocrine state.

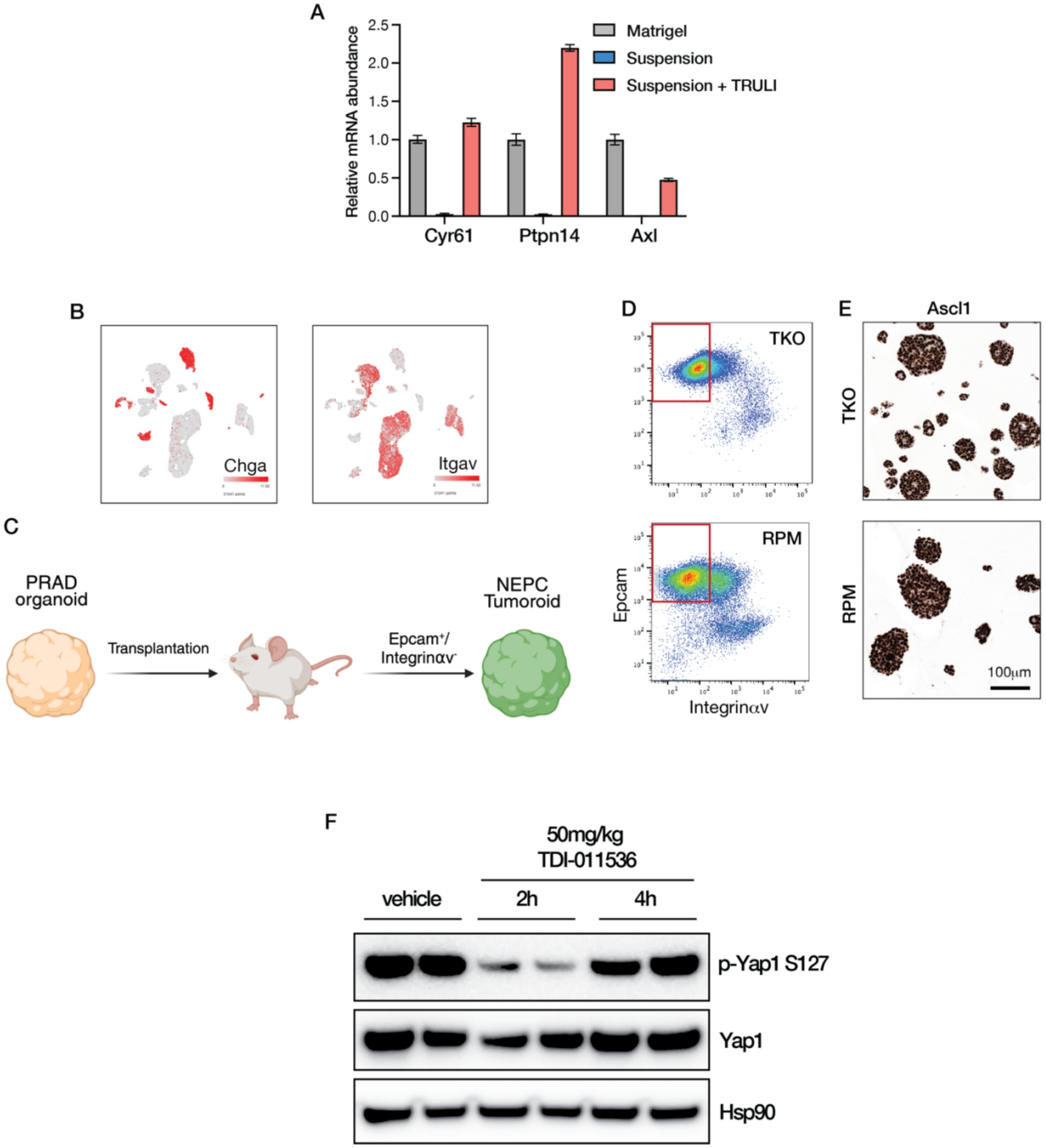

bioRxiv preprint doi: https://doi.org/10.64898/2025.12.30.697072; this version posted December 31, 2025. The copyright holder for this preprint (which was not certified by peer review) is the author/funder, who has granted bioRxiv a license to display the preprint in perpetuity. It is made available under aCC-BY 4.0 International license.

Supplemantal Figure 5. Combined inhibtion of YAP/TEAD, NOTCH and AR signaling reprograms PRAD into NEPC.

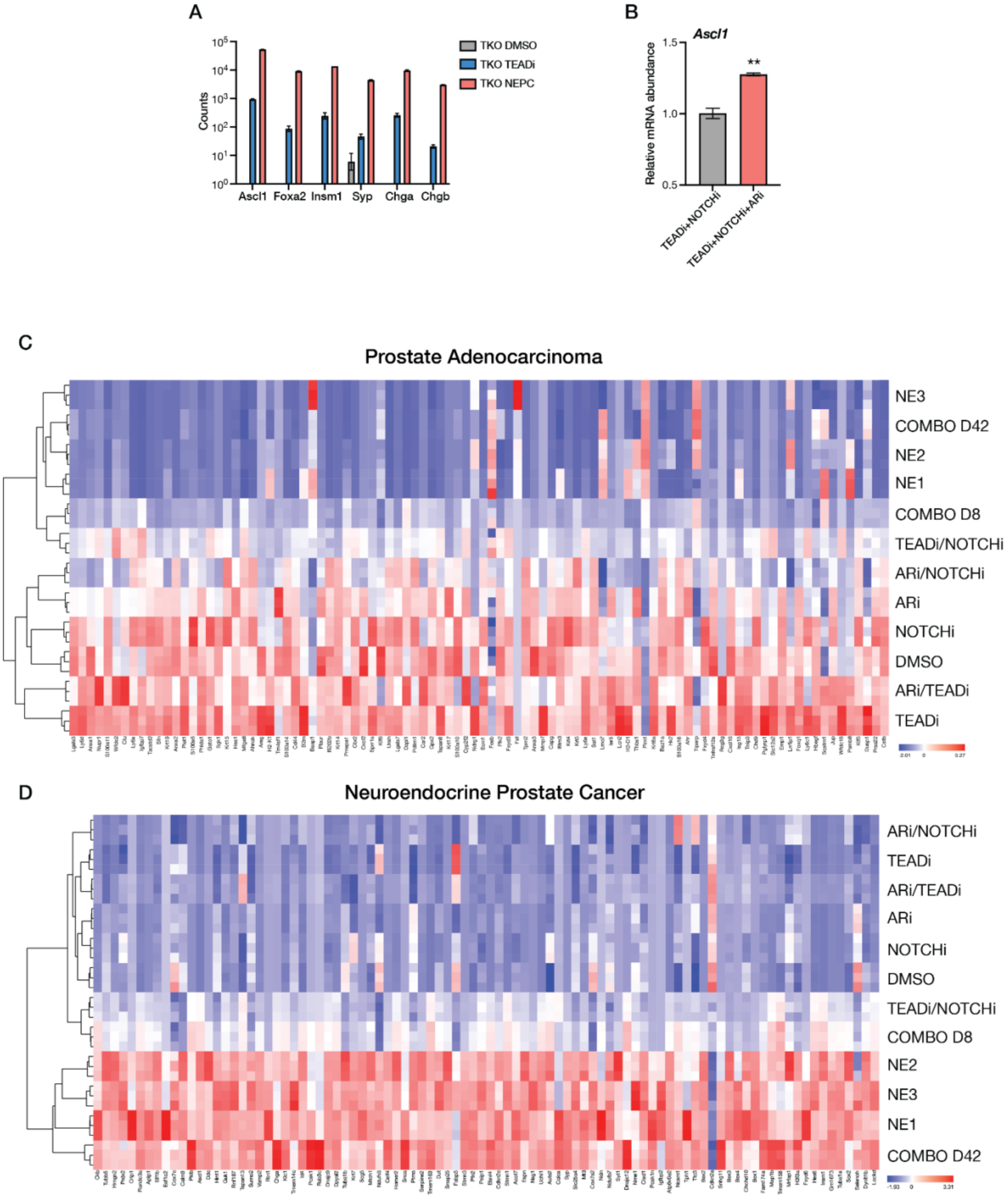

bioRxiv preprint doi: https://doi.org/10.64898/2025.12.30.697072; this version posted December 31, 2025. The copyright holder for this preprint (which was not certified by peer review) is the author/funder, who has granted bioRxiv a license to display the preprint in perpetuity. It is made available under aCC-BY 4.0 International license.

Supplemental Figure 6. TEAD and FOXA1 are essential for the PRAD to NEPC lineage transition.

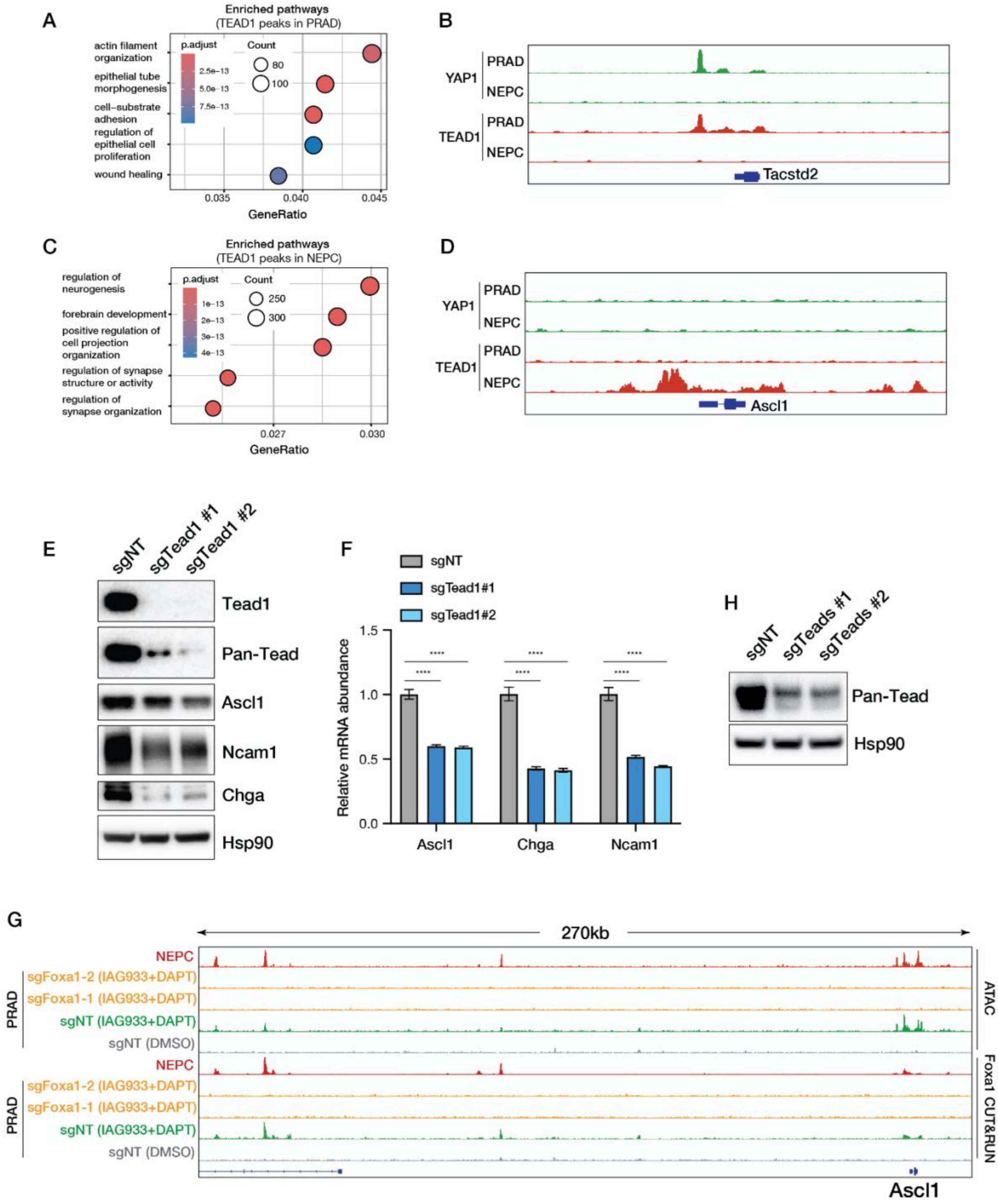

bioRxiv preprint doi: https://doi.org/10.64898/2025.12.30.697072; this version posted December 31, 2025. The copyright holder for this preprint (which was not certified by peer review) is the author/funder, who has granted bioRxiv a license to display the preprint in perpetuity. It is made available under aCC-BY 4.0 International license.

SUPPLEMENTAL FIGURE LEGENDS

Supplemental Figure 1. Cancer-associated fibroblasts and ECM are depleted in NEPC. (A) Western blot analysis confirming disruption of *Trp53*, *Rb1* and *Pten*, as well as overexpression of *cMYC* in engineered mutant organoid lines. (B) Hematoxylin and eosin staining (H&E) and Ascl1 immunohistochemistry (IHC) of TKO, RPM and TKOM organoids, as well as their corresponding orthotopic transplant tumors. (C) Vimentin IHC and Masson's trichrome staining of early- and late-stage orthotopic transplants derived from TKO and RPM organoids. (D) Deconvolved NEPC theta estimates and corresponding stromal theta estimates across eight specimens. Spatial maps highlight the distribution of NEPC and fibroblast-like content across Visium spots.

Supplemental Figure 2. Removal of extracellular matrix induces Ascl1 expression. (A) Time-course qRT-PCR analysis showing progressive induction of Ascl1 in TKOM organoids cultured under suspension conditions. Error bars represent ±SEM, n=3. (B) Heatmap displaying expression levels of NEPC transcription factors in TKOM organoids cultured in Matrigel versus suspension. (C) UMAP of scRNA-seq data from TKOM organoids under the indicated conditions. (D-G) UMAPs showing Ascl1 expression (D), Ascl1 peaks (E), Foxa2 expression (F) and Insm1 expression (G) in TKOM organoids, corresponding to the conditions in (C).

Supplemental Figure 3. YAP1/TAZ/TEAD activation blocks Ascl1 induction and PRAD to NEPC transition. (A) qRT-PCR analysis of YAP1/TAZ target genes in RPM and TKOM organoids at 6 and 10 days following Yap1 and Wwtr1 deletion via Adeno-Cre (Ad-Cre) infection. Error bars represent ±SEM, n=3. (B) qRT-PCR analysis of integrin gene expression in RPM and TKOM organoids at 6 and 10 days following Yap1 and Wwtr1 deletion via Ad-Cre. Error bars represent ±SEM, n=3. (C) qRT-PCR analysis of YAP1/TAZ target genes in RPM and TKOM organoids culturing in Matrigel, or in suspension for 14 days while overexpressing either empty vector (EV),

bioRxiv preprint doi: https://doi.org/10.64898/2025.12.30.697072; this version posted December 31, 2025. The copyright holder for this preprint (which was not certified by peer review) is the author/funder, who has granted bioRxiv a license to display the preprint in perpetuity. It is made available under aCC-BY 4.0 International license.

constitutively active YAP (YAP^{5SA}) or constitutively active TAZ (TAZ^{4SA}). Error bars represent ±SEM, n=3.

Supplemental Figure 4. LATS inhibition impairs acquisition and maintenance of neuroendocrine state. (A) qRT-PCR of YAP/TAZ target genes (*Cyr61, Ptpn14 and Axl*) in TKOM organoids cultured in Matrigel, suspension, or suspension plus 5uM TRULI for 14 days. Error bars represent ±SEM, n=3. (B) UMAP of epithelial cells in TKO GEMM tumors by scRNA-seq, showing that Itgav is silenced in NEPC cells but highly expressed in adenocarcinoma cells. NEPC cells are positive for Chga, whereas adenocarcinoma cells are Chga-negative. (C) Schematic of the workflow used to generate NEPC tumoroids from PRAD organoids. (D) Representative FACS plots showing sorting of $Epcam^{+}$/$Integrinav^{-}$ cells from TKO or RPM tumors. (E) Representative Ascl1 IHC images of organoids derived by sorting $Epcam^{+}$/$Integrinav^{-}$ cells from TKO or RPM orthotopic tumors. The scale bar represents 100mm. (F) Western blot analysis of phospho-Yap1 (S127) in mouse prostates following treatment with vehicle or TDI-011536 for 2 or 4 hours.

Supplemental Figure 5. Combined inhibition of YAP/TEAD, NOTCH and AR signaling reprograms PRAD into NEPC. (A) Normalized counts of indicated neuroendocrine markers in TKO PRAD organoids treated with DMSO or TEADi, and in TKO NEPC tumoroids. Error bars, ±SEM; n=3. (B) qRT-PCR analysis showing ARi promotes Ascl1 induction in the presence of TEADi and NOTCHi. Error bars, ±SEM; n=3; **p<0.01. (C) Heatmaps showing expression of prostate adenocarcinoma marker genes across the indicated organoid lines and treatment conditions. (D) Heatmaps showing expression of neuroendocrine prostate cancer marker genes across the same conditions as in (C).

Supplemental Figure 6. TEAD and FOXA1 are essential for the PRAD to NEPC lineage transition. (A and C) Pathway enrichment analysis of TEAD1 CUT&RUN peaks in PRAD (A) and

bioRxiv preprint doi: https://doi.org/10.64898/2025.12.30.697072; this version posted December 31, 2025. The copyright holder for this preprint (which was not certified by peer review) is the author/funder, who has granted bioRxiv a license to display the preprint in perpetuity. It is made available under aCC-BY 4.0 International license.

NEPC (C). Pathways are ranked by gene ratio. (B and D) Genome browser tracks showing CUT&RUN profiles of YAP1 and TEAD1 binding at the adenocarcinoma marker *Tacstd2* (TROP2) and the NE gene *Ascl1* in TKO PRAD and NEPC organoids. Screenshots were generated using Integrative Genomics Viewer (IGV). (E) Western blot analysis demonstrating reduced protein level of NE markers upon Tead1 knockout in TKO NEPC organoids. (F) qRT-PCR showing decreased expression of NEPC markers following Tead1 knockout in TKO NEPC organoids. Error bars represent ±SEM, n=3, ****$p<0.0001$. (G) Genome browser tracks showing ATAC-seq and FOXA1 CUT&RUN peaks at the *Ascl1* promoter and enhancer regions across the indicated organoid lines and treatment conditions. Screenshots were generated using IGV. (H) Western blot analysis showing partial knockout of TEAD proteins by CRISPR-Cas9 RNP in PRAD organoids.
